# Psychometric properties of four FACE-Q Aesthetics scales in patients planning and undergoing minimally invasive facial cosmetic procedures

**DOI:** 10.1007/s11136-026-04313-w

**Published:** 2026-06-15

**Authors:** Eszter Mercédesz Müller, Anna Nikl, Máté Krebs, Ákos Szabó, Péter Holló, Valentin Brodszky, Fanni Rencz, Lajos Vince Kemény

**Affiliations:** 1 Department of Dermatology, Venereology and Dermatooncology, Semmelweis University, 42 Mária Utca, Budapest, 1085 Hungary; 2 Semmelweis University Doctoral School, Károly Rácz Conservative Medicine Division, 26 Üllői Út, Budapest, 1085 Hungary; 3 Department of Health Policy, Corvinus University of Budapest, 8 Fővám Tér, Budapest, 1093 Hungary; 4 Department of Financial Accounting, Corvinus University of Budapest, 8 Fővám Tér, Budapest, 1093 Hungary; 5 Department of Physiology, Semmelweis University, 37-47 Tűzoltó Utca, Budapest, 1094 Hungary; 6 HCEMM-SU Translational Dermatology Research Group, Semmelweis University, 37-47 Tűzoltó Utca, Budapest, 1094 Hungary

**Keywords:** Minimally invasive cosmetic procedures, FACE-Q, Health-related quality of life, Psychometric properties

## Abstract

**Objectives:**

This study aimed to assess aspects of validity of four FACE-Q Aesthetics scales in a sample of patients undergoing and planning facial minimally invasive cosmetic procedures (MICPs), such as botulinum toxin, lip augmentation and soft tissue augmentation treatments.

**Methods:**

In 2023, a cross-sectional survey included 210 Hungarian women who had undergone and 147 planning facial MICPs, with similar mean ages Respondents completed four FACE-Q scales (Aging Appraisal, Appearance Distress, Early Life Impact and Age VAS), EQ-5D-5L, Rosenberg Self-Esteem Scale (RSES) and the Brief Fear of Negative Evaluation Scale-Straightforward Items (BFNE-S). Comprehensibility, ceiling/floor effects, structural validity (principal component analysis, confirmatory factor analysis), internal consistency, and construct validity (convergent, divergent, known-group validity) of the four FACE-Q scales were assessed.

**Results:**

All FACE-Q scales, except the Age VAS, showed a ceiling effect (20–28%). Appearance Distress showed strong convergent validity with RSES (r = 0.742), BFNE-S (r = − 0.702), and EQ-5D-5L anxiety/depression (r = − 0.519). Aging Appraisal and Appearance Distress scales were unidimensional, whereas Early Life Impact Scale had a three-factor structure. All four FACE-Q scales were able to differentiate between known groups of patients based on self-esteem, fear of negative evaluation and acceptance of bodily appearance. Women who had undergone procedures reported higher Aging Appraisal (72.9 vs. 63.3) and Appearance Distress (77.1 vs. 68.4) scores and felt younger (− 5.0 vs. − 2.8 years) than those planning them (*p* < 0.001 for all).

**Conclusions:**

Our findings provide initial support for the validity of the four FACE-Q scales in MICP populations, but further validation (e.g. assessment of responsiveness and test–retest reliability) is needed.

**Supplementary Information:**

The online version contains supplementary material available at https://doi.org/10.1007/s11136-026-04313-w.

## Introduction

The demand for minimally invasive cosmetic procedures (MICPs) has increased steadily in recent years [1]. In contrast with cosmetic surgery, these procedures have shorter recovery time and include nonsurgical techniques such as injectable treatments (e.g. neuromodulators, soft tissue fillers), lasers, chemical peels, microneedling and thread lifting [2, 3]. MICPs are most commonly sought by patients aged 40–45 years, and 93% of the patients are women [1]. Motivations often extend beyond enhancing physical beauty; many seek to improve their self-confidence, emotional state and social functioning [4, 5]. Several studies have shown that these procedures can improve health-related quality of life (HRQoL) [6–8]. However, patients might hesitate to openly discuss their concerns or personal goals with healthcare providers[9].

Assessing changes in HRQoL and understanding patients’ expectations has created a need for outcome measures that capture the patient’s perspective. HRQoL is commonly assessed using standardized questionnaires, which are typically categorized as either generic or condition-specific [10, 11]. Generic measures assess domains such as physical or mental health that apply across a wide range of populations and conditions. In contrast, condition-specific measures focus on aspects relevant to individuals undergoing particular procedures or living with specific conditions, and are more sensitive to detecting subtle, clinically meaningful changes [11].

As MICPs are elective treatments, assessing whether patients’ expectations and treatment goals have been met is important to providers [12]. While objective outcomes, such as wrinkle reduction or volume restoration, can document visible changes in facial appearance, they do not necessarily capture patients’ perceptions of those changes [13]. Moreover, unrealistic expectations, especially among patients with body dysmorphic disorder or other psychiatric conditions, may lead to dissatisfaction and an increased risk of legal action [14]. Therefore, patient-reported outcome measures (PROMs) assessing HRQoL are particularly important in populations undergoing MICPs. Until the early 2010s, evidence on the psychosocial impact of MICPs was limited and research mainly focused on traditional cosmetic surgery [15]. Despite the increasing popularity of MICPs and reported improvements in HRQoL, there is a gap in the literature regarding the rigor of reporting and measuring HRQoL beyond aesthetic improvements, particularly mental health outcomes (e.g. anxiety, depression) [16].

The FACE-Q™ Aesthetics is a modular PROM originally developed for patients undergoing aesthetic facial procedures, both surgical and nonsurgical (MICPs) [17]. Designed in accordance with established guidelines for instrument development [18, 19], it includes a set of independently functioning scales and checklists that measure outcomes based on the patients’ own priorities and experiences. The conceptual framework of FACE-Q Aesthetics covers four domains: facial appearance (27 scales), HRQoL (10 scales), natural (3 scales) and adverse effects (6 checklists) [20]. Various FACE-Q Aesthetics scales and checklists of the instrument have undergone psychometric testing [21–29] and are widely used in both clinical and research settings, including clinical trials [30–32]. Some of the FACE-Q components have also been validated in assessing the outcomes in the context of certain non-aesthetic procedures (e.g. surgery for facial trauma or functional problems of malignancies) [33, 34]. In 2022, several FACE-Q Aesthetics scales were formally recognized by the US Food and Drug Administration (FDA) as qualified medical device development tools, further underscoring its clinical and regulatory value [20].

Little is known about the psychometric performance of FACE-Q Aesthetics scales in other languages than the source language (English [31, 35–38]). Clinical trials often include multi-country populations, so outcome measures should perform well in other languages, similarly to the source language version [39]. However, most FACE-Q validation studies have focused on the English version, leaving clinicians and researchers without culturally adapted tools to standardize assessment of patients’ expectations and experiences. Furthermore, most existing FACE-Q validation studies have not conducted a comprehensive psychometric analysis across multiple properties.

This study aimed to address this gap by validating four selected FACE-Q Aesthetics HRQoL scales for use among Hungarian patients undergoing or planning facial MICPs: Aging Appraisal, Appearance-Related Psychosocial Distress (hereafter: Appearance Distress), Early Life Impact of Treatment (hereafter: Early Life Impact), and the Patient-Perceived Age Visual Analogue Scale (hereafter: Age VAS).

## Methods

### Study population

An online cross-sectional survey was conducted in Hungary between February and December 2023. Ethical approval for the study was obtained by the Semmelweis University Regional and Institutional Committee of Science and Research Ethics under reference no. SE/RKEB: 15/2023. The minimum sample size was determined in accordance with COSMIN recommendations, with at least 100 participants per subgroup (i.e. undergoing vs. planning groups) [40]. For the purposes of this study, MICPs were defined as the following treatments: soft tissue augmentation, botulinum toxin treatments, mesotherapy, monofilament thread therapy, cog thread lifting, facial laser resurfacing, medical-grade peeling and platelet-rich plasma treatment. Individuals were eligible to participate if they: (1) had undergone MICP within the previous two months (‘undergone group’) or planned to undergo MICP within the following two months (‘planning group’); (2) were 18 years of age or older; (3) were able to read and understand Hungarian; and (4) provided informed consent. Respondents who completed the questionnaire too quickly (under 6 min) or reported undergoing non-facial MICPs were excluded, as the selected FACE-Q scales are specific to facial MICPs. Participation was voluntary and anonymous. No financial incentives were provided. Participants were recruited via the research team's personal networks and social media groups. These groups serve as platforms for individuals to share experiences with MICPs. The questionnaire was distributed in six social media groups with membership sizes ranging from approximately 1,000 to 60,000 individuals. Additionally, the survey was shared on the social media platform of a large Hungarian women’s lifestyle magazine.

### Survey instrument

The survey consisted of four parts. The first section gathered data on general health status and HRQoL, including a 5-point excellent-to-poor general health scale, the EQ-5D-5L descriptive system and EQ Visual Analogue Scale (EQ VAS) and the skin irritation and self-confidence bolt-ons. The second part focused on MICPs, gathering data on the type, date, and localization of completed or planned MICPs. Only participants who had undergone MICPs within the past two months completed the Early Life Impact Scale. The third section, completed by all respondents, included the Aging Appraisal Scale, Appearance Distress Scale, the Age VAS, the Rosenberg Self-Esteem Scale (RSES) and the Brief Fear of Negative Evaluation Scale – Straightforward Items (BFNE-S). This section also included one item from the World Health Organization Quality of Life Brief Version (WHOQOL-BREF) on bodily appearance (question 11), the social relationships, tiredness and sleep bolt-ons for the EQ-5D-5L. The final part captured sociodemographic data and medical history. The order of the questions and instruments was fixed for all respondents. All survey questions were compulsory and had to be answered to proceed. The survey was programmed in Qualtrics (Qualtrics 2020, Provo, UT, USA). All questions were mandatory, and respondents could not proceed to the next question without answering the previous one; therefore, no missing data were present in the dataset.

### FACE-Q Aesthetics scales

The HRQoL domain of the FACE-Q Aesthetics module contains 10 independently functioning scales [24].

The Aging Appraisal Scale consists of 7 items assessing patients’ perceptions of their facial aging (e.g. looking into the mirror and in photos) [22]. Items are rated on a 4-point Likert scale: ‘definitely agree’, ‘somewhat agree’, ‘somewhat disagree’, ‘definitely disagree’. Respondents are asked to consider how they feel ‘today’ about the age their face looks [22].

The Appearance Distress Scale consists of 8 items assessing distress related to appearance, such as anxiety, stress and unhappiness. Items are rated on the same 4-point Likert scale [26]. This scale is completed without a defined recall period, as it reflects respondents’ general state.

The Early Life Impact Scale contains 12 items measuring the impact of a recent procedure by asking about the ability to do everyday activities (e.g. dining, social interactions) and statements about feelings (e.g. feeling anxious, tired). Responses are given on a 3-point Likert scale: ‘most of the time’, ‘some of the time’, ‘not at all’ [24]. Participants are asked to answer with reference to the previous 2 days.

The Age VAS is a single-item measure asking respondents to indicate how old they believe they look, relative to their actual age, on a scale ranging from − 15 to + 15 years. Negative values indicate feeling younger, while positive values indicate feeling older, and a score of 0 reflects perceiving oneself to look exactly one’s age [22]. The recall period is unspecified.

Selecting a limited number of scales helped reduce respondent burden, as the Hungarian versions are intended for use in routine clinical settings. The selected scales reflect the most commonly reported motivations for undergoing MICPs, including improving cosmetic appearance, looking younger and increasing self-confidence [5]. Accordingly, the Aging Appraisal Scale was included to capture the global perception of facial appearance, while the Age VAS was selected to assess perceived age. The Appearance Distress Scale was included to measure appearance-related psychological burden and self-confidence. In addition, for participants who had previously undergone MICPs, the Early Life Impact Scale was included to assess the perceived short-term effects of MICPs.

### Translation, cultural adaptation and comprehensibility of the FACE-Q scales

Permission to translate and use the FACE-Q scales to Hungarian was obtained from the original development team. The translation process followed the recommendations set forth by the International Society for Pharmacoeconomics and Outcomes Research (ISPOR) [41]. Initially, the scales were translated from English to Hungarian (forward translation) by two independent translators (a consultant dermatologist and a dermatology resident), both native Hungarian speakers (target language) and proficient in English (source language). Based on the two forward translations, a reconciled Hungarian version was produced. Afterward, the Hungarian version was back-translated into English by a native English-speaking person fluent in Hungarian, who was blinded to the original English version. The original English version and the back translation were checked for accuracy by the FACE-Q development team. Comprehensibility (a key element of content validity) was evaluated during the translation and cultural adaptation process. Cognitive debriefing interviews were performed with a convenience sample of five native Hungarian speakers, three of whom were planning to undergo MICPs and two had already undergone MICPs. The cognitive debriefing interviews were conducted by a cosmetic dermatologist to assess whether any instructions, items or response options required modification to improve comprehensibility. Patients were asked how they interpreted the instructions and items, whether any wording was difficult to understand and whether improvements were needed. They were also asked to explain the meaning of the response options and the differences between them and to indicate if any part of the response options was difficult to understand. After the cognitive debriefing interviews, the finalized translation was sent back to the development team for approval. The final versions of each scale were included in the online cross-sectional survey.

### Other outcome measures

#### EQ-5D-5L

The EQ-5D is a generic preference-accompanied HRQoL instrument, consisting of two parts: a descriptive system and a visual analogue scale (EQ VAS) [42–44].The EQ VAS records self-rated health on a vertical scale ranging from 0 (the worst health you can imagine) to 100 (the best health you can imagine). The descriptive system includes five dimensions—mobility, self-care, usual activities, pain/discomfort, and anxiety/depression—each with five levels of severity: no problems (1), slight problems (2), moderate problems (3), severe problems (4), and extreme problems/unable to (5). This structure defines 5^5^ = 3125 unique health states. Respondents are asked to recall their current health (‘your health today’). In this study, five additional EQ-5D-5L dimensions (‘bolt-ons’) were also administered, with data on these dimensions published elsewhere [45].

In this study, EQ-5D-5L index values were derived using the Hungarian value set [46]. Index values range from − 0.848 to 1, where 1 indicates full health and negative values represent health states considered worse than being dead. The Hungarian version of the EQ-5D-5L has previously been validated both qualitatively and quantitatively across a wide range of dermatological conditions [47–55].

#### Rosenberg Self-Esteem Scale (RSES)

The RSES is a widely used instrument for measuring self-esteem, consisting of ten items, each rated on a 4-point Likert scale (‘strongly agree’, ‘agree’, ‘disagree’, ‘strongly disagree’) [56, 57]. The total score ranges between 0 and 30, with higher scores indicating higher self-esteem, and a score below 15 representing low self-esteem [58]. The RSES has no specified recall period. The validated Hungarian version was used in this study [59].

#### Brief Fear of Negative Evaluation Scale-straightforward items (BFNE-S)

The BFNE-S is an eight-item questionnaire that assesses individuals’ concerns about being negatively judged by others. Each item is rated on a 5-point Likert scale, with response options ranging from ‘not at all characteristic of me’ (1) to ‘entirely characteristic of me’ (5). The total score ranges from 8 to 40, with higher scores indicating a greater fear of negative evaluation [60]. Like the RSES, the BFNE-S has no specified recall period. The validated Hungarian version was administered in this study [61].

#### Bodily appearance question

One item from the World Health Organization Quality of Life Brief Version (WHOQOL-BREF) was included to assess bodily appearance: ‘Are you able to accept your bodily appearance?’ [62]. The item is rated on a 5-point Likert scale (‘Not at all’, ‘A little’, ‘Moderately’, ‘Mostly’, ‘Completely’) and is answered with reference to the past two weeks.

### Statistical analysis

Psychometric evaluation followed the recommendations of the COnsensus-based Standards for the selection of health Measurement Instruments (COSMIN) [63]. The psychometric analyses were conducted for the total sample and, where appropriate, separately for the two subgroups (‘planning’ and ‘undergone’). All statistical analyses were performed using R Statistical Software (version 4.3.2; R Foundation for Statistical Computing, Vienna, Austria). A significance level of 0.05 was used throughout. The following measurement properties were evaluated in this study: comprehensibility, floor and ceiling effects, structural validity assessed using principal component analysis and confirmatory factor analysis, internal consistency and construct validity assessed through hypothesis testing (convergent, divergent and known-group validity).

### Descriptive statistics

Descriptive statistics were used to summarize the sociodemographic and clinical characteristics of the study population. Group differences between the ‘planning’ and ‘undergone’ participants were examined using χ^2^ test (with Yates’ continuity correction, where applicable) for categorical variables, and independent samples t-tests for continuous variables.

Raw item scores were summed for each multi-item FACE-Q scale and transformed using a Rasch-based Partial Credit Model and rescaled to a 0–100 range, where 0 represents the worst and 100 the best possible outcome [22, 24, 26]. This approach is recommended as the FACE-Q instruments were developed using Rasch Measurement Theory, which enables transformation of ordinal responses into interval-level measures, thereby linearizing the data and improving the interpretability and comparability of scores [64]. In line with this recommendation, for the assessment of scale-level psychometric properties (e.g. ceiling, floor, convergent and known-groups validity), these Rasch-transformed scores were used. For item-based analyses (e.g. structural validity), the raw item responses were used.

For the FACE-Q scales, EQ VAS, RSES, and BFNE-S, we reported the minimum and maximum values, means and standard deviations (SD), as well as medians and interquartile ranges (IQR). Item-level FACE-Q distributions were reported as relative frequencies.

### Ceiling and floor

For each FACE-Q scale, we computed the proportion of respondents reporting the maximum (ceiling) and minimum (floor) values. A proportion greater than 15% was considered indicative of a ceiling or floor effect [65].

### Convergent and divergent validity

Convergent and divergent validity were assessed by testing a priori hypotheses regarding the associations between the FACE-Q scales and external measures. Pearson’s correlation coefficients (r) were used for continuous variables (e.g. FACE-Q Rasch-transformed scores, EQ-5D-5L index value), and Spearman’s rank-order correlations (rₛ) were used for ordinal variables (e.g. FACE-Q items, EQ-5D-5L dimensions). Correlation strength was interpreted based on absolute values as follows: very weak (< 0.20), weak (0.20–0.39), moderate (0.40–0.59), strong (0.60–0.79), and very strong (≥ 0.80) [66].

First, weak or very weak correlations were hypothesized between the FACE-Q scales and most EQ-5D-5L dimensions, index values, and EQ VAS scores. An exception was the anxiety/depression dimension, which was expected to show at least a moderate correlation with the Appearance Distress Scale. Second, strong correlations were expected between the Appearance Distress Scale and both the RSES and BFNE-S, reflecting conceptual overlap between appearance-related psychosocial distress, self-esteem and fear of negative evaluation. Third, moderate or strong correlations were anticipated between the Early Life Impact, Aging Appraisal scales and Age VAS and both the RSES and BFNE-S.

### Structural validity

To assess structural validity, both principal component analysis (PCA) and confirmatory factor analysis (CFA) were conducted for all FACE-Q scales, except Age VAS. PCA was performed with varimax rotation. Sampling adequacy was evaluated using the Kaiser–Meyer–Olkin (KMO) statistic [67, 68] and factorability was confirmed via Bartlett’s test of sphericity [69]. KMO values above 0.50 were deemed acceptable [70], and a significant Bartlett’s test (*p* < 0.05) indicated suitability for PCA [71]. Factors were extracted based on the Kaiser’s criterion (eigenvalues > 1) [72], scree plot inspection, and parallel analysis [73]. Factor loadings were interpreted as acceptable (≥ 0.30), practically significant (≥ 0.50), and representative of a well-defined structure (≥ 0.70) [72]. Communalities (h^2^) > 0.50 were considered acceptable [74].

CFA was used to confirm the factor structure of each FACE-Q scale. Due to the ordinal nature of the data, diagonally weighted least squares estimation with polychoric correlations was applied. Model fit was assessed using comparative fit index (CFI), root mean square error of approximation (RMSEA), and standardized root mean square residual (SRMR). Acceptable model fit was defined as CFI > 0.90 and RMSEA and SRMR ≤ 0.08 [75].

### Internal consistency and discrimination

Internal consistency of each scale as a whole was evaluated using Cronbach’s alpha (α) coefficient. Cronbach’s α coefficients were calculated for each scale and item discrimination was assessed using corrected item-total correlations and Cronbach’s α if an item was deleted [76]. Cronbach’s α values above 0.80 were considered good, while values between 0.70 and 0.80 were acceptable, between 0.60 and 0.70 questionable, and below 0.60 poor [77]. Internal consistency was assessed only for factors with three or more items.

### Known-groups validity

Known-groups validity was assessed by comparing mean FACE-Q scale scores across subgroups a priori expected to differ. Participants were grouped by treatment status (planning vs. undergone MICPs), self-esteem (RSES < 15 vs. ≥ 15), fear of negative evaluation (BFNE-S < 25 vs. ≥ 25), and responses to the WHOQOL-BREF bodily appearance question.

Mean differences were tested using independent samples t-test (two categories) or analysis of variance (three or more categories). Effect sizes were calculated using Cohen’s d for two groups comparisons and eta-squared (η^2^) for three or more groups. Effect sizes were interpreted as negligible (d < 0.2 or η^2^ < 0.01), small (0.2 ≤ d < 0.5 or 0.01 ≤ η^2^ < 0.06), medium (0.5 ≤ d < 0.8 or 0.06 ≤ η^2^ < 0.14), and large (0.8 ≤ d or 0.14 ≤ η^2^) [78].

We hypothesized that participants who had undergone MICPs reported higher self-esteem, lower fear of negative evaluation, or greater acceptance of their appearance would score more favorably on the FACE-Q scales, i.e. lower appearance distress, more positive aging appraisal, and a perceived age equal to or lower than their actual age.

## Results

### Characteristics of the study population

The final analytic sample consisted of 357 women, of whom 210 had recently undergone and 147 were planning MICPs (Table 1). Of the 942 individuals who opened the questionnaire, 387 completed it (response rate: 41.0%). To ensure a homogeneous study population, men (n = 16) and nonbinary (n = 2) respondents were excluded. Furthermore, in line with the exclusion criteria, ‘speeders’ (i.e. those who completed the survey too quickly, n = 5) and others who reported undergoing non-facial MICP(s) (n = 7) were also excluded from the analysis.

**Table 1:** Characteristics of the study population

Variables	Total sample (n = 357)		Group 1 (undergone) (n = 210)		Group 2 (planning) (n = 147)		*p*-value^d^
	Mean or *n*	SD or %	Mean or *n*	SD or %	Mean or *n*	SD or %	
*Age (years)*							
19–24	11	3.1	6	2.9	5	3.4	0.378
25–34	104	29.1	59	28.1	45	30.6	
35–44	82	23.0	53	25.2	29	19.7	
45–54	103	28.9	59	28.1	44	29.9	
55–64	45	12.6	29	13.8	16	10.9	
65 +	12	3.4	4	1.9	8	5.4	
*Place of residence*							
Capital	178	49.9	111	52.9	67	45.6	0.053
County town	81	22.7	37	17.6	44	29.9	
Other town	68	19.0	44	21.0	24	16.3	
Village	30	8.4	18	8.6	12	8.2	
*Highest level of education*							
Primary or secondary school	86	24.1	53	25.2	33	22.4	0.521
College/university	271	75.9	157	74.8	114	77.6	
*Employment*^a^							
Full-time/self employed	286	80.1	173	82.4	113	76.9	0.015
Other	71	19.9	37	17.6	34	23.1	
*Body mass index (BMI) (kg/m*^*2*^*)*^b^							
Underweight (below 18.5)	22	6.2	16	7.6	6	4.1	0.106
Normal (between 18.5 and 24.9)	223	62.5	137	65.2	86	58.5	
Overweight (between 25 and 29.9)	61	17.1	35	16.7	26	17.7	
Obese (30 or over)	19	5.3	8	3.8	11	7.5	
*Type of procedure(s) performed in the last two months or planned in the next two months*^*c*^							
Lip augmentation	94	26.3	44	21.0	50	34.0	0.003
Soft tissue augmentation of other part of the face	81	22.7	43	20.5	38	25.9	0.164
Mesotherapy	55	15.4	27	12.9	28	19.0	0.070
Botulinum toxin treatment	159	44.5	97	46.2	62	42.2	0.401
Fine thread lifting	20	5.6	4	1.9	16	10.9	< 0.001
Cog thread lifting	35	9.8	9	4.3	26	17.7	< 0.001
Facial laser resurfacing	55	15.4	18	8.6	37	25.2	< 0.001
Medical grade peeling	21	5.9	6	2.9	15	10.2	0.002
Platelet rich plasma treatment	7	2.0	0	0.0	7	4.8	< 0.001
Other	19	5.3	14	6.7	5	3.4	0.118
*Localisation of the procedure performed in the last two months or planned in the next two months*^c^							
Upper third of the face	221	61.9	138	65.7	83	56.5	0.076
Middle third of the face	149	41.7	88	41.9	61	41.5	0.939
Lower third of the face	184	51.5	93	44.3	91	61.9	0.001
Other	15	4.2	9	4.3	6	4.1	0.925
*Adverse reaction(s) observed after the latest procedure*^c^							
No adverse reaction	–	–	139	66.2	–	–	–
Heamatoma	–	–	41	19.5	–	–	–
Pain	–	–	27	12.9	–	–	–
Oedema	–	–	31	14.8	–	–	–
Other	–	–	31	14.8	–	–	–
*Number of procedures in the last two months*							
1	–	–	155	73.8	–	–	–
2	–	–	45	21.4	–	–	
More than 2	–	–	10	4.8	–	–	
*Date of the last procedure*							
Within one week	–	–	28	13.3	–	–	–
Within two weeks	–	–	31	14.8	–	–	
Within one month	–	–	37	17.6	–	–	
Within two months	–	–	114	54.3	–	–	
*Total number of procedures performed during the lifetime*							
1–2	–	–	61	29.0	–	–	–
3–5	–	–	70	33.3	–	–	
6–9	–	–	42	20.0	–	–	
10–15	–	–	21	10.0	–	–	
16–20	–	–	3	1.4	–	–	
More than 20	–	–	13	6.2	–	–	
*Number of chronic diseases*^b^							
0	141	39.5	89	42.4	52	35.4	0.578
1	129	36.1	73	34.8	56	38.1	
2–3	75	21.0	42	20.0	33	22.4	
4	12	3.4	6	2.9	6	4.1	
*Most common chronic conditions*^c^							
Allergies	69	19.3	41	19.5	28	19.0	0.911
Thyroid disease	62	17.4	32	15.2	30	20.4	0.204
Hypertension	35	9.8	16	7.6	19	12.9	0.097
Anxiety	26	7.3	17	8.1	9	6.1	0.480
Atopic dermatitis	17	4.8	7	3.3	10	6.8	0.130
Rheumatic disease	16	4.5	11	5.2	5	3.4	0.409
Diabetes	16	4.5	8	3.8	8	5.4	0.463
Asthma or chronic obstructive pulmonary disease	16	4.5	11	5.2	5	3.4	0.409
Other dermatologic disease	14	3.9	8	3.8	6	4.1	0.896
Depression	12	3.4	6	2.9	6	4.1	0.528
Cardiovascular disease	10	2.8	5	2.4	5	3.4	0.565
Other disease	42	11.8	22	10.5	20	13.6	0.366

^a^The ‘other’ category of employment status includes part-time employed, unemployed, retired, homemaker and student

^b^The number of respondents who responded do not know and/or refused to answer was n = 22 (6.2%) for the body mass index (BMI) and n = 8 (2.2%) for the diagnosis of any chronic disease

^c^Respondents could choose more than one

^d^Pearson’s χ^2^ (with Yates’ continuity correction, where applicable) was used for categorical variables

The mean age of the total sample was 42.3 years (SD = 11.5, range: 19–71), with no significant age difference between the ‘undergone’ (mean = 42.2, SD = 10.9) and ‘planning’ (mean = 42.5, SD = 12.4) groups (Table 1). The two subsamples did not differ significantly in sociodemographic characteristics, including education level, residence or employment status. In the ‘undergone’ group, the most frequent MICPs were botulinum toxin treatment (46.2%), lip augmentation (21.0%), and soft tissue augmentation (20.5%). In the ‘planning’ group, participants most often planned botulinum toxin treatment (42.2%), lip augmentation (34.0%), and laser resurfacing (25.2%).

#### Comprehensibility

The cognitive debriefing interviews indicated that the instructions, items and response options were understandable in the context of MICPs. One small modification to the initial Hungarian version of the Age VAS was required: the first sentence was revised to emphasize ‘how old you believe you look’ rather than ‘how old you look’. Moreover, the labels of the Likert scales for the Aging Appraisal and Appearance Distress scales were slightly revised to improve clarity and distinguishability of the response levels in Hungarian. The original response options (‘not at all agree’, ‘slightly disagree’, ‘slightly agree’, ‘completely agree’) were reworded for clarity (‘definitely disagree’, ‘somewhat disagree’, ‘somewhat agree’, ‘definitely agree’) (literal back-translation from Hungarian to English).

### Outcome measures and group comparisons

**Fig. 1:**
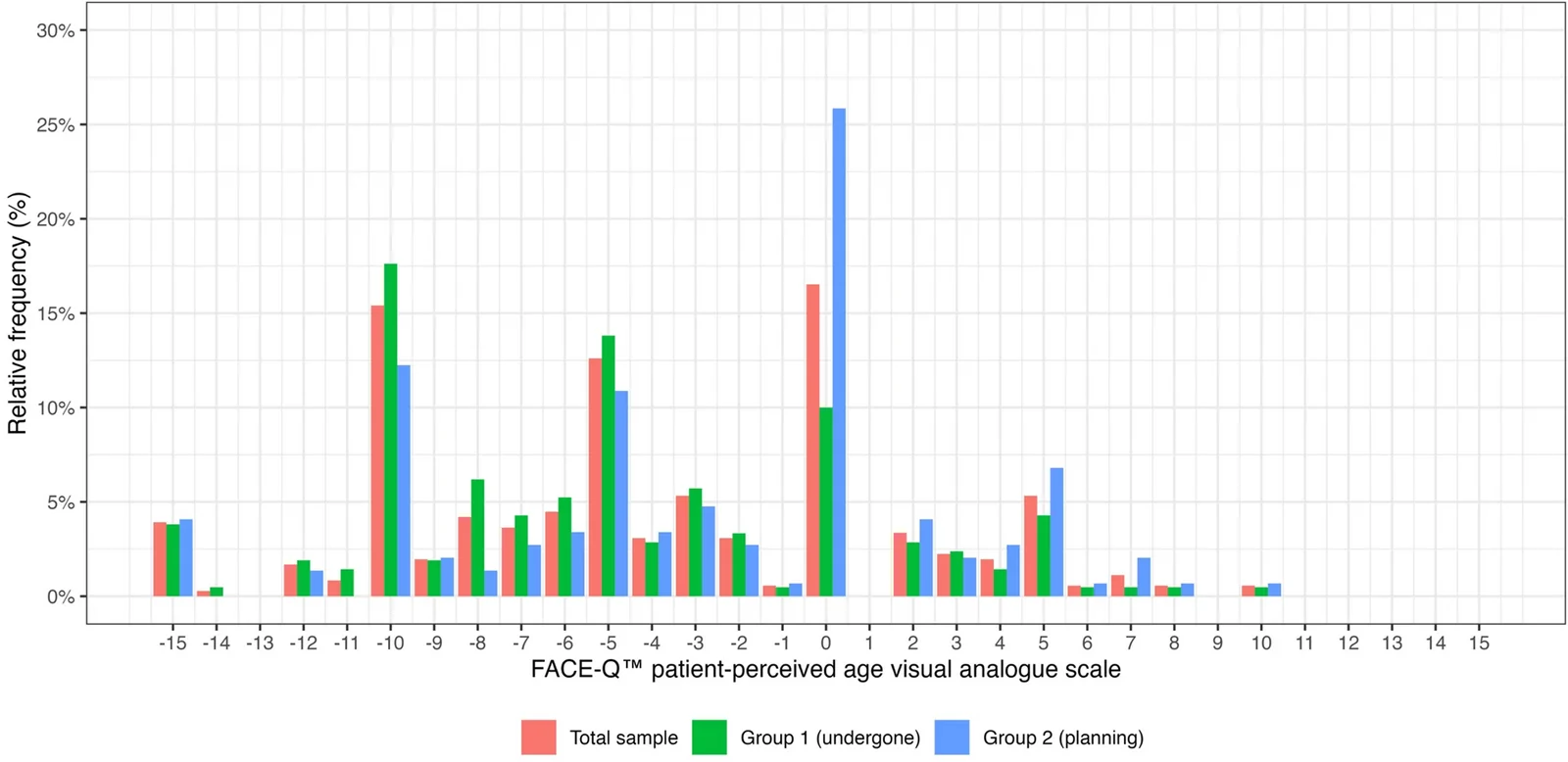
Distribution of FACE-Q Age VAS

Table 2 summarizes the descriptive statistics of the FACE-Q scales, EQ-5D-5L index values, EQ VAS, BFNE-S and RSES by treatment status. Compared to the ‘planning’ group, participants who had undergone reported more favorable scores on the FACE-Q Aging Appraisal (72.90 vs. 63.26; *p* < 0.001) and Appearance Distress Scale (77.13 vs. 68.44; *p* < 0.001) and perceived themselves as looking younger than their actual age (− 4.97 vs. − 2.81 years; *p* < 0.001). They also showed slightly better general HRQoL as indicated by the EQ-5D-5L index values (0.96 vs. 0.93; p = 0.017) and EQ VAS scores (86.57 vs. 83.57; p = 0.032), as well as lower fear of negative evaluation (17.02 vs. 20.42; *p* < 0.001), and higher self-esteem (21.99 vs. 19.88; p = 0.003). Among participants who had already undergone MICPs, the Early Life Impact Scale yielded a mean score of 75.29 (SD = 20.67). Figure 1 illustrates the distribution of perceived age differences across the total sample and by subgroup. While responses in the ‘planning’ group clustered around zero, those in the ‘undergone’ group more frequently reported feeling 5 to 10 years younger than their chronological age.

**Table 2:** Descriptive statistics of FACE–Q scales and other outcome measures

	Theoretical range	Total sample (n = 357)				Group 1. (undergone, n = 210)				Group 2. (planning, n = 147)				Group 1 mean vs Group 2 mean
		Min (%)	Max (%)	Mean (SD)	Median (IQR)	Min (%)	Max (%)	Mean (SD)	Median (IQR)	Min (%)	Max (%)	Mean (SD)	Median (IQR)	independent sample’s t-test *p*-value
FACE-Q Early Life Impact	0 to 100	–	–	–	–	1 (0.5%)	59 (28.1%)	75.29 (20.67)	71.01 (37.29)	–	–	–	–	–
FACE-Q Aging Appraisal	0 to 100	4 (1.2%)	71 (19.9%)	68.93 (23.10)	68.01 (38.08)	1 (0.5%)	51 (24.3%)	72.90 (22.02)	71.89 (32.34)	3 (2.0%)	20 (13.6%)	63.26 (23.48)	61.37 (32.40)	< 0.001
FACE-Q Appearance Distress	0 to 100	3 (0.8%)	93 (26.1%)	73.55 (21.90)	72.80 (40.97)	2 (1.0%)	65 (31.0%)	77.13 (21.01)	76.89 (36.18)	1 (0.7%)	28 (19.1%)	68.44 (22.19)	66.47 (36.22)	< 0.001
FACE-Q Age VAS	− 15 to 15	14 (3.9%)	0 (0.0%)	− 4.06 (5.53)	− 5 (9)	8 (3.8%)	0 (0.0%)	− 4.97 (5.31)	− 5 (9)	6 (4.1%)	0 (0.0%)	− 2.81 (5.60)	− 2 (6.25)	0.219
EQ-5D-5L index values	− 0.848 to 1	0 (0.0%)	170 (47.6%)	0.95 (0.10)	0.96 (0.08)	0 (0.0%)	114 (54.3%)	0.96 (0.09)	1 (0.04)	0 (0.0%)	56 (38.1%)	0.93 (0.11)	0.96 (0.08)	0.017
EQ VAS	0 to 100	0 (0.0%)	35 (9.8%)	85.33 (12.46)	90 (14)	0 (0.0%)	21 (10.0%)	86.57 (11.15)	90 (12.75)	0 (0.0%)	14 (9.5%)	83.57 (13.97)	89 (16)	0.032
Brief version of the Fear of Negative Evaluation Scale—Straightforward Items (BFNE-S)	8 to 40	25 (7.0%)	4 (1.1%)	18.42 (8.59)	16 (11)	18 (8.6%)	1 (0.5%)	17.02 (7.70)	15 (9)	7 (4.8%)	3 (2.0%)	20.42 (9.39)	18 (15)	< 0.001
Rosenberg Self-Esteem Scale (RSES)	0 to 30	1 (0.3%)	36 (10.1%)	21.12 (6.32)	22 (10)	1 (0.5%)	19 (9.1%)	21.99 (5.76)	22 (7.75)	0 (0.0%)	17 (11.6%)	19.88 (6.88)	20 (12)	0.003

FACE-Qscale scores were calculated using a Rasch-based Partial Credit Model, rescaled to a 0–100 range (0 = worst, 100 = best)

SD,  Standard deviation; IQR,  Interquartile range

### Ceiling and floor effects

In the total sample, 19.9% of participants achieved the maximum possible score on the Aging Appraisal Scale and 26.1% on the Appearance Distress Scale, while 47.6% reached the maximum on the EQ-5D-5L index value and 9.8% on the EQ VAS. In the’undergone’ group, the proportions of participants achieving the maximum score were 24.3% for Aging Appraisal Scale, 31.0% for Appearance Distress Scale, 54.3% for the EQ-5D-5L index value and 10.0% for the EQ VAS. In the’planning’ group, the corresponding proportions were 13.6%, 19.1%, 38.1%, and 9.5%, respectively (Table 2). None of the four FACE-Q scales showed floor effects across any subgroup.

### Convergent and divergent validity

In line with our hypothesis, the FACE-Q scales showed weak or very weak correlations with EQ-5D-5L dimensions, index values, and EQ VAS (Table 3). The Appearance Distress Scale demonstrated a moderate correlation with EQ-5D-5L anxiety/depression (r_s_ = − 0.519) and with the EQ VAS (r_s_ = 0.410). This scale also showed strong correlations with both the RSES (r = 0.742) and BFNE-S total scores (r = − 0.702).

**Table 3:** Convergent and divergent validity of the FACE-Q scale scores

	FACE-Q early life impact scale	FACE-Q aging appraisal scale	FACE-Q appearance distress	FACE-Q age VAS
*EQ-5D-5L*				
Mobility	− 0.066*	− 0.181	− 0.108	− 0.032*
Self-care	− 0.075*	− 0.073*	− 0.054*	− 0.071*
Usual activities	− 0.138	− 0.232	− 0.277	0.114
Pain/discomfort	− 0.114*	− 0.185	− 0.328	0.142
Anxiety/depression	− 0.225	− 0.185	− 0.519	0.223
EQ-5D-5L index values	0.170	0.262	0.372	− 0.101*
EQ VAS scores	0.248	0.311	0.410	− 0.155
Rosenberg Self-Esteem Scale	0.213	0.320	0.742	− 0.423
Brief Fear of Negative Evaluation Scale—Straightforward Items	− 0.248	− 0.359	− 0.702	0.415

Pearson’s correlation was used for continuous variables, and Spearman’s rank-order correlation for ordinal dimensions

*Correlation coefficient p-value ≥ 0.05

As anticipated, most items from the Early Life Impact and Aging Appraisal scales showed weak or very weak correlations with EQ-5D-5L dimensions, except for six items of the Appearance Distress Scale, which moderately correlated with the EQ-5D-5L anxiety/depression dimension (Table 4).

**Table 4:** Convergent and divergent validity of the EQ-5D-5L dimensions

Items	EQ-5D-5L				
	Mobility	Self-care	Usual activities	Pain/discomfort	Anxiety/depression
*FACE-Q Early Life Impact—items*					
Item 1—Regret	0.054*	0.013*	0.045*	0.056*	− 0.013*
Item 2—Anxious	− 0.087*	− 0.193	− 0.188	− 0.170	− 0.167
Item 3—Sleeping	− 0.141	− 0.122*	− 0.168	− 0.123*	− 0.145*
Item 4—Worthwhile	0.018*	0.032*	− 0.007*	− 0.096*	0.006*
Item 5—Tired	0.044*	0.020*	− 0.162	− 0.062*	− 0.146*
Item 6—Head movements	0.062*	0.044*	0.068*	0.093*	− 0.097*
Item 7—Usual activities	− 0.010*	0.024*	− 0.113*	0.038*	− 0.002*
Item 8—Facial movements	0.035*	0.047*	− 0.024*	0.053*	− 0.096*
Item 9—Drinking	0.006*	0.020*	− 0.047*	− 0.087*	− 0.204
Item 10—Eating	− 0.026*	0.017*	− 0.088*	− 0.166	− 0.187
Item 11—Social situations	− 0.092*	0.030*	− 0.118*	0.009*	− 0.126
Item 12—Intimacy	− 0.101*	− 0.198	− 0.201	− 0.098*	− 0.123*
*FACE-Q appearance distress items*					
Item 1—Feel unhappy	− 0.062*	− 0.011*	− 0.199	− 0.273	− 0.413
Item 2—Feel stressed	− 0.098*	− 0.010*	− 0.171	− 0.244	− 0.387
Item 3—Feel down	− 0.082*	0.041*	− 0.222	− 0.317	− 0.423
Item 4—Feel anxious	− 0.046*	− 0.001*	− 0.181	− 0.276	− 0.458
Item 5—Look normal	− 0.089*	− 0.015*	− 0.123	− 0.158	− 0.331
Item 6—Ugly	− 0.050*	0.008*	− 0.132	− 0.217	− 0.441
Item 7—Avoid people	− 0.073*	− 0.077*	− 0.265	− 0.289	− 0.462
Item 8—Doing things	− 0.119	− 0.078*	− 0.304	− 0.340	− 0.417
*FACE-Q aging appraisal—items*					
Item 1—Don’t recognize	− 0.192	− 0.092*	− 0.297	− 0.188	− 0.194
Item 2—Look in mirror	− 0.162	− 0.059*	− 0.313	− 0.305	− 0.330
Item 3—Bothered	− 0.226	− 0.043*	− 0.216	− 0.195	− 0.165
Item 4—Older	− 0.107	0.007*	− 0.207	− 0.172	− 0.153
Item 5—Worried	− 0.130	− 0.064*	− 0.160	− 0.124	− 0.113
Item 6—Photos	− 0.149	− 0.033*	− 0.212	− 0.197	− 0.182
Item 7—Reflection	− 0.143	− 0.027*	− 0.211	− 0.150	− 0.172

Spearman’s rank-order correlation was used to assess associations between individual FACE-Q scale items and EQ-5D-5L dimensions

* Correlation coefficient *p*-value ≥ 0.05

### Principal component analysis

PCA identified a four-factor structure for the Early Life Impact Scale, with most items loading onto one of the four factors. Item 5 and item 7 showed lower factor loadings, while item 11 cross-loaded on two factors. In contrast, the Aging Appraisal and Appearance Distress Scale demonstrated clear unidimensionality, with consistently high factor loadings and communalities across all samples. Factor loadings ranged from 0.67 to 0.87 for the Aging Appraisal Scale and from 0.55 to 0.88 for the Appearance Distress Scale in the total sample. One item showed relatively weaker performance on each scale across all samples: item 2 on the Aging Appraisal and item 8 on the Appearance Distress Scale, both showing the lowest factor loadings and communalities in their respective scales (Table 5).

**Table 5:** Results of the principal component analysis of the FACE-Q scales

	Total sample (n = 357)		Group 1. (undergone, n = 210)					Group 2. (planning, n = 147)	
	Factor loadings	Communalities (h^2^)	Factor 1 loadings ‘Social and functional difficulties’	Factor 2 loadings ‘Post-procedure regret and anxiety’	Factor 3 loadings ‘Movement and activity limitations’	Factor 4 loadings ‘Sleep disturbance and fatigue’	Communalities (h^2^)	Factor loadings	Communalities (h^2^)
*FACE-Q Early Life Impact—items*									
Item 1—Regret	**–**	**–**		0.610			0.373	**–**	**–**
Item 2—Anxious	**–**	**–**		0.429			0.234	**–**	**–**
Item 3—Sleeping	**–**	**–**				0.602	0.482	**–**	**–**
Item 4—Worthwhile	**–**	**–**		0.596			0.372	**–**	**–**
Item 5—Tired	**–**	**–**				0.465	0.227	**–**	**–**
Item 6—Head movements	**–**	**–**			0.633		0.405	**–**	**–**
Item 7—Usual activities	**–**	**–**			0.407		0.324	**–**	**–**
Item 8—Facial movements	**–**	**–**			0.686		0.569	**–**	**–**
Item 9—Drinking	**–**	**–**	0.776				0.624	**–**	**–**
Item 10—Eating	**–**	**–**	0.780				0.684	**–**	**–**
Item 11—Social situations	**–**	**–**	0.434	0.409			0.436	**–**	**–**
Item 12—Intimacy	**–**	**–**	0.647				0.582	**–**	**–**
Kaiser**–**Meyer**–**Olkin measure (KMO)	**–**	**–**	0.708					**–**	**–**
Bartlett’s test	**–**	**–**	*χ*^2^ = 586.35 (*df* = 66), * p* < 0.001					**–**	**–**

	Factor loadings	Communalities (h^2^)	Factor loadings	Communalities (h^2^)	Factor loadings	Communalities (h^2^)
*FACE-Q Aging Appraisal—items*						
Item 1—Don’t recognize	0.726	0.527	0.687	0.472	0.748	0.559
Item 2—Look in mirror	0.672	0.452	0.722	0.521	0.593	0.352
Item 3—Bothered	0.872	0.760	0.841	0.707	0.899	0.808
Item 4—Older	0.805	0.649	0.767	0.588	0.834	0.696
Item 5—Worried	0.765	0.586	0.765	0.585	0.765	0.586
Item 6—Photos	0.838	0.701	0.819	0.671	0.845	0.714
Item 7—Reflection	0.870	0.757	0.867	0.752	0.876	0.767
Kaiser–Meyer**–**Olkin measure (KMO)	0.899		0.891		0.883	
Bartlett’s test	*χ*^2^ = 1819.66 (*df* = 21), * p* < 0.001		*χ*^2^ = 1005.35 (*df* = 21), * p* < 0.001		*χ*^2^ = 785.64 (*df* = 21), * p* < 0.001	
*FACE-Q Appearance Distress—items*						
Item 1—Feel unhappy	0.842	0.710	0.813	0.661	0.865	0.749
Item 2—Feel stressed	0.823	0.678	0.812	0.659	0.825	0.681
Item 3—Feel down	0.848	0.718	0.849	0.721	0.835	0.697
Item 4—Feel anxious	0.881	0.776	0.850	0.723	0.908	0.825
Item 5—Look normal	0.746	0.557	0.724	0.525	0.753	0.567
Item 6—Ugly	0.830	0.689	0.806	0.650	0.841	0.707
Item 7—Avoid people	0.710	0.504	0.712	0.506	0.688	0.474
Item 8—Doing things	0.546	0.298	0.540	0.291	0.523	0.273
Kaiser**–**Meyer**–**Olkin measure (KMO)	0.917		0.907		0.908	
Bartlett’s test	*χ*^2^ = 2103.99 (*df* = 28), * p* < 0.001		*χ*^2^ = 1129.61 (*df* = 28), * p* < 0.001		*χ*^2^ = 904.71 (*df* = 28), * p* < 0.001	

Factor loadings > 0.40 are shown in the table

### Confirmatory factor analysis

CFA generally supported the factorial structure of FACE-Q scales (Table 6). For the Early Life Impact Scale, the four-factor model could not be tested because one factor comprised only two items; therefore, a three-factor model (excluding items 3 and 5) was evaluated. In the ‘undergone’ group, model fit indices were CFI = 0.988, RMSEA = 0.050, and SRMR = 0.118. For the Aging Appraisal and Appearance Distress scales, CFI values ranged from 0.996 to 0.998 and SRMR from 0.056 to 0.069, while RMSEA values ranged from 0.071 to 0.130, with the highest values observed in the ‘planning’ group (0.108–0.130).

**Table 6:** Results of the confirmatory factor analysis

	CFI	RMSEA	SRMR
*FACE-Q Early Life Impact*			
Group 1 (undergone, n = 210)			
Three-factor model excluding items 3 and 5	**0.988**	**0.050**	0.118
*FACE-Q Aging Appraisal*			
Total sample (n = 357)			
One-factor model including all items	**0.996**	0.118	**0.058**
Group 1 (undergone, n = 210)			
One-factor model including all items	**0.997**	0.097	**0.063**
Group 2 (planning, n = 147)			
One-factor model including all items	**0.996**	0.130	**0.063**
*FACE-Q Appearance Distress Distress*			
Total sample (n = 357)			
One-factor model including all items	**0.997**	0.095	**0.058**
Group 1 (undergone, n = 210)			
One-factor model including all items	**0.998**	**0.071**	**0.056**
Group 2 (planning, n = 147)			
One-factor model including all items	**0.997**	0.108	**0.069**

Recommended values: CFI > 0.90, RMSEA and SRMR ≤ 0.08. Values meeting the cut-off criteria are indicated in bold

CFI comparative fit index, RMSEA root mean square error of approximation, SRMR standardized root mean square residual

### Internal consistency and discrimination

Cronbach’s α coefficients for the Early Life Impact Scale ranged from 0.605 to 0.754 across factors (Table 7). The highest internal consistency was observed for Factor 1 (α = 0.754), while Factors 2 and 3 showed lower values (α = 0.605 and α = 0.658, respectively). Corrected item-total correlations ranged from 0.224 to 0.638.

**Table 7:** Internal consistency and item statistics of the Early Life Impact scale

FACE-Q™ early life impact—items	Group 1. (undergone, n = 210)			
	Factor 1 Cronbach’s alpha (if item removed)	Factor 2 Cronbach’s alpha (if item removed)	Factor 3 Cronbach’s alpha (if item removed)	Discrimination (corrected item-total correlation)
Item 1—Regret	–	0.557	–	0.323
Item 2—Anxious	–	0.547	–	0.366
Item 3—Sleeping	–	0.556	–	0.382
Item 4—Worthwhile	–	0.517	–	0.348
Item 5—Tired	–	–	–	0.224
Item 6—Head movements	–	–	0.626	0.287
Item 7—Usual activities	–	–	0.628	0.544
Item 8—Facial movements	–	–	0.482	0.477
Item 9—Drinking	0.684	–	–	0.591
Item 10—Eating	0.719	–	–	0.587
Item 11—Social situations	0.717	0.573	–	0.638
Item 12—Intimacy	0.651	–	0.609	0.626
*Scale*	0.754	0.605	0.658	–

In contrast, the Aging Appraisal and Appearance Distress scales demonstrated excellent internal consistency across the total sample and subgroups (α = 0.913–0.926). Corrected item-total correlations ranged from 0.546 to 0.899 (Table 8).

**Table 8:** Internal consistency and item statistics of the aging appraisal and appearance distress scales

	Total sample (n = 357)		Group 1. (undergone, n = 210)		Group 2. (planning, n = 147)	
	Cronbach’s alpha (if item removed)	Discrimination (corrected item-total correlation)	Cronbach’s alpha (if item removed)	Discrimination (corrected item-total correlation)	Cronbach’s alpha (if item removed)	Discrimination (corrected item-total correlation)
*FACE-Q™ aging appraisal*—*items*						
Item 1—Don’t recognize	0.916	0.741	0.911	0.709	0.914	0.760
Item 2—Look in mirror	0.919	0.688	0.907	0.739	0.928	0.608
Item 3—Bothered	0.901	0.862	0.894	0.832	0.900	0.890
Item 4—Older	0.907	0.797	0.901	0.758	0.906	0.831
Item 5—Worried	0.911	0.768	0.902	0.764	0.912	0.772
Item 6—Photos	0.903	0.838	0.895	0.818	0.904	0.848
Item 7—Reflection	0.900	0.863	0.890	0.858	0.901	0.867
*Scale*	0.920	–	0.913	–	0.922	–
*FACE-Q™ appearance distress*—*items*						
Item 1—Feel unhappy	0.912	0.840	0.904	0.810	0.911	0.863
Item 2—Feel stressed	0.913	0.821	0.905	0.811	0.914	0.820
Item 3—Feel down	0.911	0.845	0.902	0.846	0.913	0.833
Item 4—Feel anxious	0.908	0.875	0.901	0.849	0.907	0.899
Item 5—Look normal	0.918	0.748	0.911	0.730	0.918	0.753
Item 6—Ugly	0.912	0.822	0.905	0.797	0.912	0.835
Item 7—Avoid people	0.921	0.727	0.912	0.727	0.922	0.713
Item 8—Doing things	0.931	0.559	0.923	0.548	0.933	0.546
*Scale*	0.926	–	0.919	–	0.926	–

### Known-group validity

As hypothesized, participants who had undergone MICPs reported significantly more favorable outcomes on the FACE-Q scales than those planning MICPs (Table 9). Similarly, participants with higher self-esteem (RSES ≥ 15) also scored significantly better on all FACE-Q scales and felt younger, while those with greater fear of negative evaluation (BFNE-S ≥ 25) had significantly lower scores across all FACE-Q scales and, on average, perceived themselves as looking older. Scores on all four FACE-Q scales also increased with higher levels of acceptance of bodily appearance. Based on effect sizes, the Appearance Distress Scale showed three large and one small effect, followed by the Aging Appraisal Scale and Age VAS (each showing one large, two medium, and one small), while the Early Life Impact Scale showed one medium and two small effects.

**Table 9:** Known-groups validity of four FACE-Q scales

	n (%) (‘undergone’ sample)	FACE-Q early life impact			n (%) (pooled sample)	FACE-Q aging appraisal			FACE-Q APPEARANCE DISTREss			FACE-Q age VAS		
		Mean (SD)	*p*-value^a^	Effect size^b^		Mean (SD)	*p*-value^a^	Effect size ^*b*^	Mean (SD)	*p*-value^a^	Effect size^b^	Mean (SD)	*p*-value^a^	Effect size^b^
Subgroups														
Group 1. (undergone)	210 (100.0%)	75.29 (20.67)	–	–	210 (58.8%)	72.90 (22.02)	< 0.001	0.427	77.13 (21.01)	< 0.001	0.405	–4.97 (5.31)	< 0.001	0.219
Group 2. (planning)	–	–			147 (41.2%)	63.26 (23.48)			68.44 (22.19)			–2.81 (5.60)		
Total score on the Rosenberg Self–Esteem Scale (RSE)														
Less than 15	22 (10.5%)	66.37 (18.32)	0.025	0.488	60 (16.8%)	57.06 (25.32)	< 0.001	0.636	46.16 (17.42)	< 0.001	1.822	− 0.42 (4.98)	< 0.001	0.653
At least 15	188 (89.5%)	76.34 (20.72)			297 (83.2%)	71.33 (21.90)			79.09 (18.26)			− 4.78 (5.35)		
Total score on the Brief Fear of Negative Evaluation Scale-Straightforward Items (BFNE-S)														
< 25	176 (83.8%)	76.80 (20.0)	0.031	0.459	279 (78.2%)	72.2 (21.6)	< 0.001	0.670	80.51 (16.8)	< 0.001	1.825	− 4.94 (5.21)	< 0.001	0.773
≥ 25	34 (16.2%)	67.57 (22.8)			78 (21.8%)	57.3 (24.5)			48.65 (19.7)			− 0.88 (5.52)		
Are you able to accept your bodily appearance?														
Not at all	10 (4.8%)	64.73 (28.90)	0.010	0.062	16 (4.5%)	44.43 (22.98)	< 0.001	0.179	34.63 (23.52)	< 0.001	0.513	− 0.33 (7.50)	< 0.001	0.213
A little	9 (4.3%)	60.71 (14.79)			31 (8.7%)	51.45 (23.71)			46.85 (12.57)			2.41 (3.53)		
Moderately	38 (18.1%)	69.69 (19.39)			81 (22.7%)	65.09 (23.77)			62.54 (16.38)			− 3.03 (4.75)		
Mostly	35 (16.7%)	78.22 (20.59)			183 (51.3%)	71.61 (20.33)			80.93 (15.81)			− 5.11 (5.13)		
Completely	118 (56.2%)	78.27 (17.99)			46 (12.9%)	85.32 (15.47)			95.09 (8.43)			− 7.4 (4.05)		

The FACE-Q Early Life Impact scale was completed only by participants in the ‘undergone’ group (n = 210), while the other scales were administered across the pooled sample (n = 357)

^a^Difference in mean scores between groups was tested by independent sample’s t-test (two categories) or analysis of variance (three or more categories)

^b^Effect sizes were calculated using Cohen's d (two categories) or η^2^ (three or more categories)

## Discussion

In this study we provided initial support for validity and reliability four FACE-Q Aesthetics scales (Aging Appraisal, Appearance Distress, Early Life Impact, and the Age VAS) among Hungarian women planning and undergoing MICPs. Despite small ceiling effects in three of the four scales, all scales demonstrated acceptable psychometric performance, with strong evidence for construct and known-groups validity, particularly for the Appearance Distress Scale. This scale showed strong correlation with external measures of self-esteem, fear of negative evaluation, anxiety and depression. Aging Appraisal and Appearance Distress scales demonstrated a unidimensional structure, whereas the Early Life Impact Scale showed a three-factor structure. Regarding reliability, the Early Life Impact Scale showed variable reliability, with acceptable internal consistency for Factor 1 and questionable values for Factors 2–3. In contrast, the Aging Appraisal and Appearance Distress scales demonstrated high reliability and strong item–total correlations, indicating more consistent measurement. In addition, all four FACE-Q scales differentiated between groups defined by self-esteem, fear of negative evaluation, and acceptance of bodily appearance. Women who had undergone MICPs reported higher Aging Appraisal and Appearance Distress scores and perceived themselves as younger compared with those planning treatment.

The four FACE-Q scales have earlier been translated into several languages [79]. However, to date, none of them have been validated in populations undergoing MICPs. Validation studies using the FACE-Q instrument in aesthetic populations have primarily focused on procedure-specific modules, particularly in surgical contexts. Several cross-cultural adaptations have been reported, including validation studies of the German FACE-Q Eye module in blepharoplasty patients [80], rhinoplasty modules in Dutch [81], Finnish [82] and French [83] populations, and the Satisfaction with Facial Appearance Overall scale in Brazilian rhytidoplasty patients [84]. These studies reported acceptable psychometric properties, including internal consistency, reliability and construct validity across different cultural settings.

We were able to directly compare the’planning’ and’undergone’ groups, as they were well matched on key sociodemographics, including age, education, and residence. As hypothesized, women who had undergone MICPs reported more favorable outcomes than those planning such procedures: they experienced significantly lower appearance distress, more positive appraisals of aging, and perceived themselves younger. This finding reinforces previous research suggesting that the pursuit of a more youthful appearance is a key motivation for undergoing MICPs [5] and that individuals undergoing such procedures often report favorable HRQoL and self-esteem outcomes [85]. However, the results should be interpreted considering the cross-sectional design of the study.

The Early Life Impact, Aging Appraisal, and Appearance Distress scales showed notable ceiling effects in the group that had undergone MICPs, whereas in the ‘planning’ group, only the Appearance Distress Scale demonstrated a ceiling effect. Ceiling effects may be expected in MICP populations, where post-treatment satisfaction and psychosocial scores are often high following successful MICPs [12]. These effects may limit the ability of the scales to distinguish between respondents with very high scores and to detect further improvement over time in longitudinal assessments. It is also worth noting that body dysmorphic disorder may be present in approximately 10% of patients in cosmetic dermatology practices and these patients may report greater dissatisfaction with their appearance [86].

A particular strength of our study is the combined use of the EQ-5D-5L and FACE-Q Aesthetics in the same survey, which has not been investigated before. In terms of construct validity, correlations with most EQ-5D-5L dimensions were weak, which may reflect not only the limited sensitivity of generic HRQoL instruments in capturing the specific effects of MICPs, but also differences in the constructs assessed by generic and appearance-specific instruments. The EQ-5D-5L [42, 44] primarily focuses on generic HRQoL dimensions (e.g. mobility, pain/discomfort), whereas appearance-specific scales such as Psychosocial Distress and Aging Appraisal reflect appearance-related psychosocial functioning. Our previous study found that adding relevant bolt-ons (e.g. self-confidence) substantially enhanced the psychometric performance of the EQ-5D-5L in this population [45]. By contrast, the Appearance Distress Scale correlated moderately with the EQ-5D-5L anxiety/depression dimension and the EQ VAS and strongly with both the RSES and BFNE-S. This pattern highlights the conceptual overlap between appearance-related distress and broader psychosocial problems such as self-esteem, fear of negative evaluation, anxiety and depression [87–90]. Importantly, patients experiencing elevated appearance-related distress may remain dissatisfied despite objectively successful treatment outcomes, a conclusion supported by evidence from skin cancer populations where pre-existing anxiety or depression predicted greater postoperative appearance distress [91].

Factor analysis indicated a single-factor structure for Aging Appraisal and Appearance Distress scales, with all items loading on a single factor. This is consistent with the structure reported for the original instrument [22, 26]. The Early Life Impact scale showed a more complex factor structure, PCA yielded a four-factor solution rather than a single factor, while CFA supported a three-factor model (excluding the two-item Factor 4). The identified factors reflected different aspects of the early post-procedure period, including social and functional difficulties, post-procedure regret and anxiety, movement and activity limitations. This suggests that early treatment impact following MICPs may not represent a single underlying domain. This divergence may reflect differences in the nature of MICPs compared to surgical procedures (such as facelift surgery and minimally invasive lip treatments) studied in earlier validation studies [24]. MICPs include a wide range of procedures with different recovery patterns and adverse events. Injectable procedures, such as botulinum toxin treatment and soft tissue fillers, are commonly associated with early local reactions including pain, oedema, erythema and ecchymosis, while rare severe early complications include vascular occlusion and necrosis. In contrast, adverse events following procedures such as chemical peels may develop over subsequent weeks and include prolonged erythema, delayed wound healing, dyspigmentation and scarring [92]. This variability in early post-procedure experiences may contribute to the complex factor structure observed for the Early Life Impact scale and may also suggest cultural variation in how Hungarian patients conceptualize post-treatment impacts, with a clearer distinction between social and physical domains of experience.

Clinically, the validated Hungarian FACE-Q scales have several applications. Their use in cosmetic dermatology practice could facilitate pre-treatment counseling by clarifying patient expectations and identifying those at risk of heightened appearance distress. Their systematic use in follow-up may also help monitor outcomes, align clinical results with patient satisfaction, and reduce the risk of unmet expectations [26]. From a research perspective, these scales enable standardized outcome measurement in Hungarian populations, and their availability opens the way for cross-national comparisons.

This study has several limitations. First, convenience sampling and inclusion of only women may limit the representativeness of the sample, reducing generalizability to men, older adults, or other demographic subgroups. Although MICPs are more commonly performed among women, the number of male patients undergoing MICPs is increasing [1], and further validation in more gender-diverse populations is needed. Second, the study was not conducted in a clinical setting, data were collected via a self-administered online survey, relying on self-reported clinical information that was not verified by physicians. The results reflect this specific online sample, which may be subject to self-selection bias and may not be generalizable to the wider MICPs populations in Hungary or beyond. Third, the fixed order of standardized questionnaires may have introduced order effects. Fourth, content validity assessment in this study was limited to comprehensibility as part of the cognitive debriefing after the translations and a small sample. Therefore, evaluation of other important aspects, including relevance and comprehensiveness of the FACE-Q scales in larger MICP samples, is recommended in the future. Moreover, the included FACE-Q scales use different recall periods, which may have influenced participants’ responses. Finally, given the cross-sectional design of this study, test–retest reliability, measurement error, and responsiveness were not assessed, which also represents a limitation of the comparison between the’undergone’ and’planning’ groups. Future studies using longitudinal designs could further evaluate these measurement properties. Cross-cultural validity (e.g. measurement invariance by language) was also not assessed in this study, therefore, equivalence with the original English version cannot yet be assumed. However, a clear strength of the study was the ability to compare the ‘planning’ and ‘undergone’ groups, as well as the validation conducted in a language other than English and in a context different from previous validation studies, which were mainly carried out in Western countries.

In conclusion, this is the first psychometric assessment of four FACE-Q Aesthetics scales in the context of facial MICPs, supporting their preliminary use in both clinical and research settings. The different factor structure of the Early Life Impact Scale requires further investigation in diverse populations. While the results provide initial support for the validity of the Hungarian FACE-Q HRQoL scales, further validation work (e.g. assessment of responsiveness and test–retest reliability) is needed before these four scales can be considered fully validated. Routine application of FACE-Q could enhance patient-centered care in cosmetic dermatology and contribute to a more nuanced understanding of psychosocial outcomes of MICPs.

## Supplementary Information

Below is the link to the electronic supplementary material.

Supplementary Material 1

## Data Availability

All data of this study are available from the corresponding author upon reasonable request.

## Code availability

N/A.

## References

[1] American Society of Plastic Surgeons. (2024). Plastic surgery statistics report. Retrieved March 2, 2025, from https://www.plasticsurgery.org/documents/news/statistics/2024/plastic-surgery-statistics-report-2024.pdf

[2] Devgan, L., Singh, P., & Durairaj, K. (2019). Minimally invasive facial cosmetic procedures. *Otolaryngologic Clinics of North America,**52*, 443–459. 10.1016/j.otc.2019.02.013 30954270

[3] Chuang, J., Barnes, C., & Wong, B. J. F. (2016). Overview of facial plastic surgery and current developments. *The Surgery Journal,**2*, e17-28. 10.1055/s-0036-1572360 28824978 PMC5553462

[4] Waldman, A., Maisel, A., Weil, A., Iyengar, S., Sacotte, K., Lazaroff, J. M., et al. (2019). Patients believe that cosmetic procedures affect their quality of life: An interview study of patient-reported motivations. *Journal of the American Academy of Dermatology,**80*, 1671–1681. 10.1016/j.jaad.2019.01.059 30710607

[5] Maisel, A., Waldman, A., Furlan, K., Weil, A., Sacotte, K., Lazaroff, J. M., et al. (2018). Self-reported patient motivations for seeking cosmetic procedures. *JAMA Dermatology,**154*, 1167–1174. 10.1001/jamadermatol.2018.2357 30140900 PMC6233736

[6] Ribeiro, F., & Steiner, D. (2018). Quality of life before and after cosmetic procedures on the face: A cross-sectional study in a public service. *Journal of Cosmetic Dermatology,**17*, 688–692. 10.1111/jocd.12723 30105787

[7] Rudolph, C., Hladik, C., Stroup, D. F., Frank, K., Gotkin, R. H., Dayan, S. H., et al. (2019). Are cosmetic procedures comparable to antidepressive medication for quality-of-life improvements? A systematic review and controlled meta-analysis. *Facial Plastic Surgery,**35*, 549–558. 10.1055/s-0039-1697030 31563125

[8] McKeown, D. J. (2021). Impact of minimally invasive aesthetic procedures on the psychological and social dimensions of health. *Plastic and Reconstructive—Surgery Global Open,**9*, e3578. 10.1097/GOX.0000000000003578 33936919 PMC8081460

[9] Werschler, W. P., Calkin, J. M., Laub, D. A., Mauricio, T., Narurkar, V. A., & Rich, P. (2015). Aesthetic dermatologic treatments: Consensus from the experts. *The Journal of Clinical and Aesthetic Dermatology,**8*, S2-7. 26587179 PMC4635431

[10] Jackowski, D., & Guyatt, G. (2003). A guide to health measurement. *Clinical Orthopaedics and Related Research*. 10.1097/01.blo.0000079771.06654.13 12897599

[11] Patrick, D. L., & Deyo, R. A. (1989). Generic and disease-specific measures in assessing health status and quality of life. *Medical Care,**27*, S217-232. 10.1097/00005650-198903001-00018 2646490

[12] Hoffman, L., & Fabi, S. (2022). Look better, feel better, live better? The impact of minimally invasive aesthetic procedures on satisfaction with appearance and psychosocial wellbeing. *The Journal of Clinical and Aesthetic Dermatology,**15*, 47–58. 35642226 PMC9122280

[13] Sobanko, J. F., Dai, J., Gelfand, J. M., Sarwer, D. B., & Percec, I. (2018). Prospective cohort study investigating changes in body image, quality of life, and self-esteem following minimally invasive cosmetic procedures. *Dermatologic Surgery,**44*, 1121–1128. 10.1097/DSS.0000000000001523 29659404

[14] Crerand, C. E., Menard, W., & Phillips, K. A. (2010). Surgical and minimally invasive cosmetic procedures among persons with body dysmorphic disorder. *Annals of plastic surgery,**65*, 11–16. 10.1097/SAP.0b013e3181bba08f 20467296 PMC3083632

[15] Imadojemu, S., Sarwer, D. B., Percec, I., Sonnad, S. S., Goldsack, J. E., Berman, M., et al. (2013). Influence of surgical and minimally invasive facial cosmetic procedures on psychosocial outcomes: A systematic review. *JAMA Dermatology,**149*, 1325–1333. 10.1001/jamadermatol.2013.6812 24068036

[16] Hemsworth, B., Hemsworth, C., & Richmond, S. A. (2024). Nonsurgical medical aesthetics and patient quality of life: An umbrella review. *Aesthetic Surgery Journal Open Forum,**6*, ojae096. 10.1093/asjof/ojae096 39678691 PMC11646117

[17] Klassen, A. F., Cano, S. J., Scott, A., Snell, L., & Pusic, A. L. (2010). Measuring patient-reported outcomes in facial aesthetic patients: Development of the FACE-Q. *Facial Plastic Surgery,**26*, 303–309. 10.1055/s-0030-1262313 20665408

[18] Aaronson, N., Alonso, J., Burnam, A., Lohr, K., Patrick, D. L., Perrin, E., et al. (2002). Scientific Advisory Committee of Medical Outcomes Trust. Assessing health status and quality of life instruments: Attributes and review criteria. *Quality of Life Research,**11*, 193–205. 12074258 10.1023/a:1015291021312

[19] U.S. Department of Health and Human Services FDA Center for Drug Evaluation and Research, U.S. Department of Health and Human Services FDA Center for Biologics Evaluation and Research, U.S. Department of Health and Human Services FDA Center for Devices and Radiological Health. Guidance for industry: patient-reported outcome measures: use in medical product development to support labeling claims: draft guidance. Health and Quality of Life Outcomes 2006;4:79. 10.1186/1477-7525-4-79 PMC1629006 17034633

[20] Q-Portfolio. (2022). FACE-Q® | Aesthetics: A user’s guide for researchers and clinicians. Retrieved May 24, 2025, from https://qportfolio.org/wp-content/uploads/2023/01/FACE-Q-AESTHETICS-USERSGUIDE.pdf

[21] Pusic, A. L., Klassen, A. F., Scott, A. M., & Cano, S. J. (2013). Development and psychometric evaluation of the FACE-Q satisfaction with appearance scale: A new patient-reported outcome instrument for facial aesthetics patients. *Clinics in Plastic Surgery,**40*, 249–260. 10.1016/j.cps.2012.12.001 23506765

[22] Panchapakesan, V., Klassen, A. F., Cano, S. J., Scott, A. M., & Pusic, A. L. (2013). Development and psychometric evaluation of the FACE-Q aging appraisal scale and patient-perceived age visual analog scale. *Aesthetic Surgery Journal,**33*, 1099–1109. 10.1177/1090820X13510170 24243890

[23] Klassen, A. F., Cano, S. J., Scott, A. M., & Pusic, A. L. (2014). Measuring outcomes that matter to face-lift patients: Development and validation of FACE-Q appearance appraisal scales and adverse effects checklist for the lower face and neck. *Plastic and Reconstructive Surgery,**133*, 21–30. 10.1097/01.prs.0000436814.11462.94 24105086

[24] Klassen, A. F., Cano, S. J., Schwitzer, J. A., Scott, A. M., & Pusic, A. L. (2015). FACE-Q scales for health-related quality of life, early life impact, satisfaction with outcomes, and decision to have treatment: Development and validation. *Plastic and Reconstructive Surgery,**135*, 375–386. 10.1097/PRS.0000000000000895 25626785

[25] Klassen, A. F., Cano, S. J., East, C. A., Baker, S. B., Badia, L., Schwitzer, J. A., et al. (2016). Development and psychometric evaluation of the FACE-Q scales for patients undergoing rhinoplasty. *JAMA Facial Plastic Surgery,**18*, 27–35. 10.1001/jamafacial.2015.1445 26605889 PMC4831065

[26] Klassen, A. F., Cano, S. J., Alderman, A., East, C., Badia, L., Baker, S. B., et al. (2016). Self-report scales to measure expectations and appearance-related psychosocial distress in patients seeking cosmetic treatments. *Aesthetic Surgery Journal,**36*, 1068–1078. 10.1093/asj/sjw078 27222106 PMC5029370

[27] Klassen, A. F., Cano, S. J., Schwitzer, J. A., Baker, S. B., Carruthers, A., Carruthers, J., et al. (2016). Development and psychometric validation of the FACE-Q skin, lips, and facial rhytids appearance scales and adverse effects checklists for cosmetic procedures. *JAMA Dermatology,**152*, 443–451. 10.1001/jamadermatol.2016.0018 26934294 PMC4833666

[28] Klassen, A. F., Cano, S. J., Grotting, J. C., Baker, S. B., Carruthers, J., Carruthers, A., et al. (2017). FACE-Q eye module for measuring patient-reported outcomes following cosmetic eye treatments. *JAMA Facial Plastic Surgery,**19*, 7–14. 10.1001/jamafacial.2016.1018 27631534 PMC5247311

[29] Schwitzer, J. A., Klassen, A. F., Cano, S. J., Baker, S. B., East, C., & Pusic, A. L. (2015). Measuring satisfaction with appearance: Validation of the FACE-Q scales for the nose, forehead, cheekbones, and chin. *Plastic and Reconstructive Surgery,**136*, 140. 10.1097/01.prs.0000472460.10389.65

[30] Gallo, L., Kim, P., Churchill, I. F., Gallo, M., Yuan, M., Voineskos, S. H., et al. (2024). Measuring the impact of surgical and non-surgical facial cosmetic interventions using FACE-Q aesthetic module scales: A systematic review and meta-analysis. *Plastic Surgery*. 10.1177/22925503231225480 39553513 PMC11562317

[31] Ottenhof, M. J., Veldhuizen, I. J., Hensbergen, L. J. V., Blankensteijn, L. L., Bramer, W., Lei, B. V., et al. (2022). The use of the FACE-Q aesthetic: A narrative review. *Aesthetic Plastic Surgery,**46*, 2769–2780. 10.1007/s00266-022-02974-9 35764813 PMC9729314

[32] Ascher, B., Rzany, B., Kestemont, P., Hilton, S., Heckmann, M., Bodokh, I., et al. (2020). Significantly increased patient satisfaction following liquid formulation AbobotulinumtoxinA treatment in glabellar lines: FACE-Q outcomes from a phase 3 clinical trial. *Aesthetic Surgery Journal,**40*, 1000–1008. 10.1093/asj/sjz248 31550352 PMC7427150

[33] Homsy, S. P., Repo, J. P., Lindford, A. J., Uimonen, M. M., & Lassus, P. (2022). Validation of the Finnish FACE-Q for use in patients undergoing surgery for functional problems or malignancy. *Journal of Plastic Surgery and Hand Surgery,**56*, 270–276. 10.1080/2000656X.2021.1964981 34428115

[34] Elegbede, A., Mermulla, S., Diaconu, S. C., McNichols, C., Liang, Y., Liang, F., et al. (2018). Patient-reported outcomes in facial reconstruction: Assessment of FACE-Q scales and predictors of satisfaction. *Plastic and Reconstructive Surgery Global Open,**6*, Article e2004. 10.1097/GOX.0000000000002004 30656106 PMC6326617

[35] Bustillo, A. M. B., Lobato, R. C., Luitgards, B. F., Camargo, C. P., Gemperli, R., & Ishida, L. C. (2019). Translation, cross-cultural adaptation and linguistic validation of the FACE-Q questionnaire for Brazilian Portuguese. *Aesthetic Plastic Surgery,**43*, 930–937. 10.1007/s00266-019-01399-1 31089752

[36] Homsy, P., Uimonen, M., Lindford, A., Repo, J., & Lassus, P. (2021). Finnish translation and validation of the FACE-Q Eye module. *Scandinavian Journal of Surgery,**110*, 504–511. 10.1177/1457496920982767 33372569

[37] Sadok, N., Mischke, M., Bastian, T., Mattheis, S., Lang, S., & Stähr, K. (2025). German translation and validation of the FACE-Q nose and nostrils subscales. *Plastic and Reconstructive Surgery. Global Open,**13*, Article e7021. 10.1097/GOX.0000000000007021 40757379 PMC12316326

[38] Ottenhof, M. J., Dobbs, T. D., Veldhuizen, I., Harrison, C. J., Marges, M., Lee, E. H., et al. (2024). FACE-Q for measuring patient-reported outcomes after facial skin cancer surgery: Cross-cultural validation. *Plastic and Reconstructive Surgery. Global Open,**12*, Article e5771. 10.1097/GOX.0000000000005771 38689944 PMC11057807

[39] Prakash, V., Shah, S., & Hariohm, K. (2019). Cross-cultural adaptation of patient-reported outcome measures: A solution or a problem? *Annals of Physical and Rehabilitation Medicine,**62*, 174–177. 10.1016/j.rehab.2019.01.006 30753895

[40] Mokkink, L. B., Elsman, E. B. M., & Terwee, C. B. (2024). COSMIN guideline for systematic reviews of patient-reported outcome measures version 2.0. *Quality of Life Research: An International Journal of Quality of Life Aspects of Treatment, Care and Rehabilitation,**33*, 2929–2939. 10.1007/s11136-024-03761-6 39198348 PMC11541334

[41] Wild, D., Grove, A., Martin, M., Eremenco, S., McElroy, S., Verjee-Lorenz, A., et al. (2005). Principles of good practice for the translation and cultural adaptation process for patient-reported outcomes (PRO) measures: Report of the ISPOR task force for translation and cultural adaptation. *Value in Health,**8*, 94–104. 10.1111/j.1524-4733.2005.04054.x 15804318

[42] Brooks, R. (1996). EuroQol: The current state of play. *Health Policy (Amsterdam, Netherlands),**37*, 53–72. 10.1016/0168-8510(96)00822-6 10158943

[43] Herdman, M., Gudex, C., Lloyd, A., Janssen, M., Kind, P., Parkin, D., et al. (2011). Development and preliminary testing of the new five-level version of EQ-5D (EQ-5D-5L). *Quality of Life Research,**20*, 1727–1736. 10.1007/s11136-011-9903-x 21479777 PMC3220807

[44] EuroQol Group. (1990). EuroQol—A new facility for the measurement of health-related quality of life. *Health Policy (Amsterdam, Netherlands),**16*, 199–208. 10.1016/0168-8510(90)90421-9 10109801

[45] Müller, E. M., Nikl, A., Krebs, M., Holló, P., Brodszky, V., Kemény, L. V., et al. (2025). Psychometric benefits of adding bolt-ons to the EQ-5D-5L in populations undergoing minimally invasive cosmetic procedures. *The European Journal of Health Economics*. 10.1007/s10198-025-01772-9 40075019 PMC12432065

[46] Rencz, F., Brodszky, V., Gulácsi, L., Golicki, D., Ruzsa, G., Pickard, A. S., et al. (2020). Parallel valuation of the EQ-5D-3L and EQ-5D-5L by time trade-off in Hungary. *Value in Health,**23*, 1235–1245. 10.1016/j.jval.2020.03.019 32940242

[47] Szabó, Á., Brodszky, V., & Rencz, F. (2024). Comparing EQ-5D-5L, PROPr, SF-6D and TTO utilities in patients with chronic skin diseases. *European Journal of Health Economics*. 10.1007/s10198-024-01728-5 PMC12126321 39340749

[48] Koszorú, K., Hajdu, K., Brodszky, V., Bató, A., Gergely, L. H., Kovács, A., et al. (2023). Comparing the psychometric properties of the EQ-5D-3L and EQ-5D-5L descriptive systems and utilities in atopic dermatitis. *European Journal of Health Economics,**24*, 139–152. 10.1007/s10198-022-01460-y PMC9877050 35412162

[49] Bató, A., Brodszky, V., Gergely, L. H., Gáspár, K., Wikonkál, N., Kinyó, Á., et al. (2021). The measurement performance of the EQ-5D-5L versus EQ-5D-3L in patients with hidradenitis suppurativa. *Quality of Life Research,**30*, 1477–1490. 10.1007/s11136-020-02732-x 33534032 PMC8068690

[50] Gergely, L. H., Gáspár, K., Brodszky, V., Kinyó, Á., Szegedi, A., Remenyik, É., et al. (2020). Validity of EQ-5D-5L, Skindex-16, DLQI and DLQI-R in patients with hidradenitis suppurativa. *Journal of the European Academy of Dermatology and Venereology,**34*, 2584–2592. 10.1111/jdv.16642 32618022

[51] Tamási, B., Brodszky, V., Péntek, M., Gulácsi, L., Hajdu, K., Sárdy, M., et al. (2019). Validity of the EQ-5D in patients with pemphigus vulgaris and pemphigus foliaceus. *The British Journal of Dermatology,**180*, 802–809. 10.1111/bjd.16883 29897626

[52] Poór, A. K., Rencz, F., Brodszky, V., Gulácsi, L., Beretzky, Z., Hidvégi, B., et al. (2017). Measurement properties of the EQ-5D-5L compared to the EQ-5D-3L in psoriasis patients. *Quality of Life Research,**26*, 3409–3419. 10.1007/s11136-017-1699-x 28875430

[53] Plázár, D., Metyovinyi, Z., Medvecz, M., & Rencz, F. (2024). Qualitative evidence on EQ-5D-5L skin irritation and self-confidence bolt-ons in Darier’s disease and Hailey-Hailey disease. *Quality of Life Research*. 10.1007/s11136-024-03871-1 39704914 PMC11982107

[54] Szlávicz, E., Szabó, Á., Kinyó, Á., Szeiffert, A., Bancsók, T., Brodszky, V., et al. (2024). Content validity of the EQ-5D-5L with skin irritation and self-confidence bolt-ons in patients with atopic dermatitis: A qualitative think-aloud study. *Quality of Life Research,**33*, 101–111. 10.1007/s11136-023-03519-6 37787930 PMC10784357

[55] Rencz, F., Mukuria, C., Bató, A., Poór, A. K., & Finch, A. P. (2022). A qualitative investigation of the relevance of skin irritation and self-confidence bolt-ons and their conceptual overlap with the EQ-5D in patients with psoriasis. *Quality of Life Research,**31*, 3049–3060. 10.1007/s11136-022-03141-y 35471487 PMC9039271

[56] Rosenberg, M. (2011). Rosenberg self-esteem scale. *Journal of Religion and Health*. 10.1037/t01038-000

[57] Donnellan, M. B., Trzesniewski, K. H., & Robins, R. W. (2015). Measures of self-esteem. In G. J. Boyle, D. H. Saklofske, & G. Matthews (Eds.), *Measures of personality and social psychological constructs* (pp. 131–157). Academic Press.

[58] Isomaa, R., Väänänen, J.-M., Fröjd, S., Kaltiala, R., & Marttunen, M. (2013). How low is low? Low self-esteem as an indicator of internalizing psychopathology in adolescence. *Health Education & Behavior,**40*, 392–399. 10.1177/1090198112445481 22872582

[59] Sallay, V., Martos, T., Földvári, M., Szabó, T., & Ittzés, A. (2014). A Rosenberg Önértékelés Skála (RSES-H): Alternatív fordítás, strukturális invariancia és validitás [Hungarian version of the Rosenberg Self-esteem Scale (RSES-H): An alternative translation, structural invariance, and validity]. *Mentálhigiéné es Pszichoszomatika,**15*, 259–275. 10.1556/Mental.15.2014.3.7

[60] Rodebaugh, T. L., Woods, C. M., Thissen, D. M., Heimberg, R. G., Chambless, D. L., & Rapee, R. M. (2004). More information from fewer questions: The factor structure and item properties of the original and brief fear of negative evaluation scale. *Psychological Assessment,**16*, 169–181. 10.1037/1040-3590.16.2.169 15222813

[61] Perczel-Forintos, D., & Kresznerits, S. (2017). Social anxiety and self-esteem: Hungarian validation of the “Brief Fear of Negative Evaluation Scale—Straightforward Items.” *Orvosi Hetilap,**158*, 843–850. 10.1556/650.2017.30755 28561634

[62] Development of the World Health Organization WHOQOL-BREF quality of life assessment. The WHOQOL Group. Psychological Medicine 1998;28:551–8. 10.1017/s0033291798006667 9626712

[63] Terwee, C. B., Prinsen, C. A. C., Chiarotto, A., Westerman, M. J., Patrick, D. L., Alonso, J., Bouter, L. M., de Vet, H. C. W., & Mokkink, L. B. (2018). COSMIN methodology for evaluating the content validity of patient-reported outcome measures: A Delphi study. *Quality of Life Research,**27*(5), 1159–1170. 10.1007/s11136-018-1829-0 29550964 PMC5891557

[64] Gallo, L., Kim, P., Yuan, M., Gallo, M., Thoma, A., Voineskos, S. H., et al. (2023). Best practices for FACE-Q aesthetics research: A systematic review of study methodology. *Aesthetic Surgery Journal,**43*, 674–686. 10.1093/asj/sjad141 37162009

[65] Terwee, C. B., Bot, S. D. M., de Boer, M. R., van der Windt, D. A. W. M., Knol, D. L., Dekker, J., et al. (2007). Quality criteria were proposed for measurement properties of health status questionnaires. *Journal of Clinical Epidemiology,**60*, 34–42. 10.1016/j.jclinepi.2006.03.012 17161752

[66] Evans, J. D. (1996). *Straightforward statistics for the behavioral sciences*. Thomson Brooks/Cole.

[67] Kaiser, H. F., & Rice, J. (1974). Little Jiffy, Mark Iv. *Educational and Psychological Measurement,**34*, 111–117. 10.1177/001316447403400115

[68] Kaiser, H. F. (1970). A second generation little jiffy. *Psychometrika,**35*, 401–415. 10.1007/BF02291817

[69] Bartlett, M. S. (1950). Tests of significance in factor analysis. *British Journal of Statistical Psychology,**3*, 77–85. 10.1111/j.2044-8317.1950.tb00285.x

[70] Kaiser, H. F. (1974). An index of factorial simplicity. *Psychometrika,**39*, 31–36. 10.1007/BF02291575

[71] Tobias, S., & Carlson, J. E. (1969). Brief report: Bartlett’s test of sphericity and chance findings in factor analysis. *Multivariate Behavioral Research,**4*, 375–377. 10.1207/s15327906mbr0403_8 26745847

[72] Brown, T. A. (2015). *Confirmatory factor analysis for applied research* (2nd ed.). Guilford Press.

[73] Franklin, S. B., Gibson, D. J., Robertson, P. A., Pohlmann, J. T., & Fralish, J. S. (1995). Parallel analysis: A method for determining significant principal components. *Journal of Vegetation Science,**6*, 99–106. 10.2307/3236261

[74] Black, W. C., Babin, B. J., & Anderson, R. E. (2009). *Multivariate data analysis* (7th ed.). Pearson.

[75] Hu, L., & Bentler, P. M. (1999). Cutoff criteria for fit indexes in covariance structure analysis: Conventional criteria versus new alternatives. *Structural Equation Modeling: A Multidisciplinary Journal,**6*, 1–55. 10.1080/10705519909540118

[76] Cronbach, L. J. (1951). Coefficient alpha and the internal structure of tests. *Psychometrika,**16*, 297–334. 10.1007/BF02310555

[77] Nunnally, J. C., & Bernstein, I. H. (1994). *Psychometric theory*. McGraw-Hill.

[78] Cohen, J. (1992). A power primer. *Psychological Bulletin,**112*, 155–159. 10.1037/0033-2909.112.1.155 19565683

[79] Q-Portfolio. (2025). FACE-Q® | Aesthetics: Translations. Retrieved May 24, 2025, from https://qportfolio.org/wp-content/uploads/2025/03/FACE-Q-AESTHETICS-Translations.pdf

[80] Fan, W., Dai, W., Bechtloff, C., Ju, X., Li, X., Hou, X., et al. (2024). FACE-Q eye module for measuring patient-reported outcomes in blepharoplasty surgery: A validation study. *Journal of Cranio-Maxillo-Facial Surgery: Official Publication of the European Association for Cranio-Maxillo-Facial Surgery,**52*, 1006–1011. 10.1016/j.jcms.2024.06.017 38871618

[81] Pingnet, L., Verkest, V., Fransen, E., & Declau, F. (2021). Dutch translation and validation of the FACE-Q rhinoplasty module. *Facial Plastic Surgery,**37*, 296–301. 10.1055/s-0040-1721099 33506453

[82] Homsy, S. P., Uimonen, M. M., Lindford, A. J., Repo, J. P., & Lassus, P. A. (2021). Application of the FACE-Q rhinoplasty module in a mixed reconstructive and corrective rhinoplasty population in Finland. *Journal of Plastic Surgery and Hand Surgery,**55*, 373–379. 10.1080/2000656X.2021.1898973 33729899

[83] Radulesco, T., Penicaud, M., Santini, L., Graziani, J., Dessi, P., & Michel, J. (2019). French validation of the FACE-Q rhinoplasty module. *Clinical Otolaryngology,**44*, 240–243. 10.1111/coa.13267 30506633

[84] Gama, J. T., Rossetto, L. A., Brito, N. B., Veiga, D. F., & Ferreira, L. M. (2020). Cross-cultural validation of the FACE-Q Satisfaction with Facial Appearance Overall Scale (FACE-Q SFAOS) in Brazilian rhytidoplasty patients. *Clinics,**75*, Article e1568. 10.6061/clinics/2020/e1568 32756818 PMC7384204

[85] de Aquino, M. S., Haddad, A., & Ferreira, L. M. (2013). Assessment of quality of life in patients who underwent minimally invasive cosmetic procedures. *Aesthetic Plastic Surgery,**37*, 497–503. 10.1007/s00266-012-9992-0 23519872

[86] Veale, D., Gledhill, L. J., Christodoulou, P., & Hodsoll, J. (2016). Body dysmorphic disorder in different settings: A systematic review and estimated weighted prevalence. *Body Image,**18*, 168–186. 10.1016/j.bodyim.2016.07.003 27498379

[87] Almudimeegh, A., Almukhadeb, E., Nagshabandi, K. N., Alshehri, N., Almusa, H. A., Aldosari, O., et al. (2025). The impact of individuals’ self-esteem on cosmetic dermatology preferences assessed using the Rosenberg Self-Esteem Scale: A cross-sectional study. *Medicine,**104*, Article e43109. 10.1097/MD.0000000000043109 40587688 PMC12212790

[88] Chopra, D., & Ii, R. D. L. G. (2019). Depressive, anxiety, and distress symptoms among cancer patients who endorse appearance problems. *Palliative & Supportive Care,**17*, 328–332. 10.1017/S1478951518000494 30109833

[89] Bullen, T. L., Sharpe, L., Lawsin, C., Patel, D. C., Clarke, S., & Bokey, L. (2012). Body image as a predictor of psychopathology in surgical patients with colorectal disease. *Journal of Psychosomatic Research,**73*, 459–463. 10.1016/j.jpsychores.2012.08.010 23148815

[90] Chang, O., Choi, E.-K., Kim, I.-R., Nam, S.-J., Lee, J. E., Lee, S. K., et al. (2014). Association between socioeconomic status and altered appearance distress, body image, and quality of life among breast cancer patients. *Asian Pacific Journal of Cancer Prevention,**15*, 8607–8612. 10.7314/APJCP.2014.15.20.8607 25374176

[91] Vaidya, T. S., Mori, S., Dusza, S. W., Rossi, A. M., Nehal, K. S., & Lee, E. H. (2019). Appearance-related psychosocial distress following facial skin cancer surgery using the FACE-Q skin cancer. *Archives of Dermatological Research,**311*, 691–696. 10.1007/s00403-019-01957-2 31338583 PMC7474556

[92] Levy, L. L., & Emer, J. J. (2012). Complications of minimally invasive cosmetic procedures: prevention and management. *Journal of Cutaneous and Aesthetic Surgery,**5*, 121–132. 10.4103/0974-2077.99451 23060707 PMC3461789

