# Supplementary material for: Psychometric properties of four FACE-Q Aesthetics scales in patients planning and undergoing minimally invasive facial cosmetic procedures
Source: Qual Life Res. 2026 Jun 15;35(8):201. doi: 10.1007/s11136-026-04313-w (PMC13269543; doi:10.1007/s11136-026-04313-w)

**Online resources**

**Online resource 1. Distribution of the responses on the FACE-Q Early Life Impact scale**


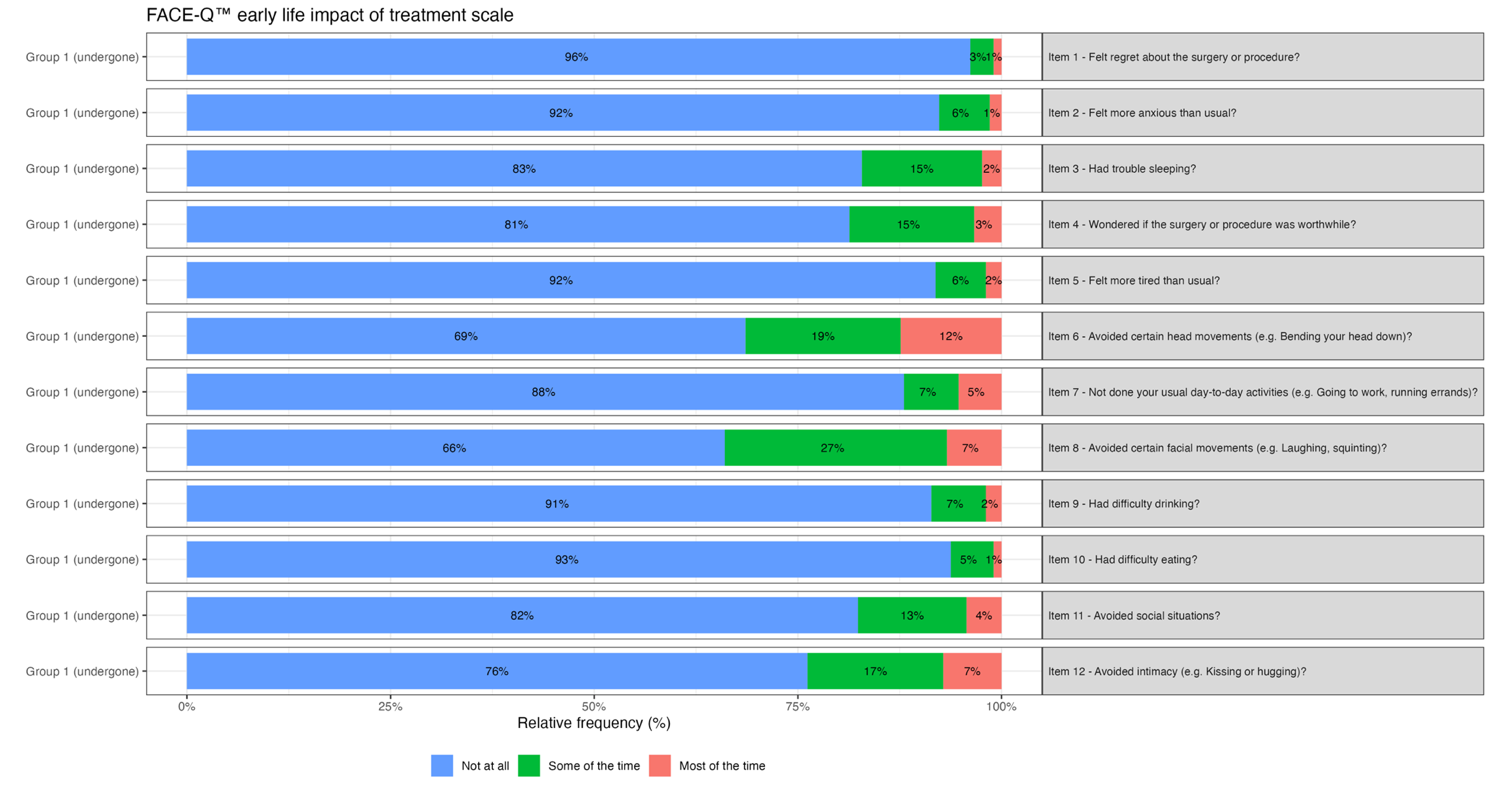


**Online resource 2. Distribution of the responses on the FACE-Q Aging Appraisal Scale**


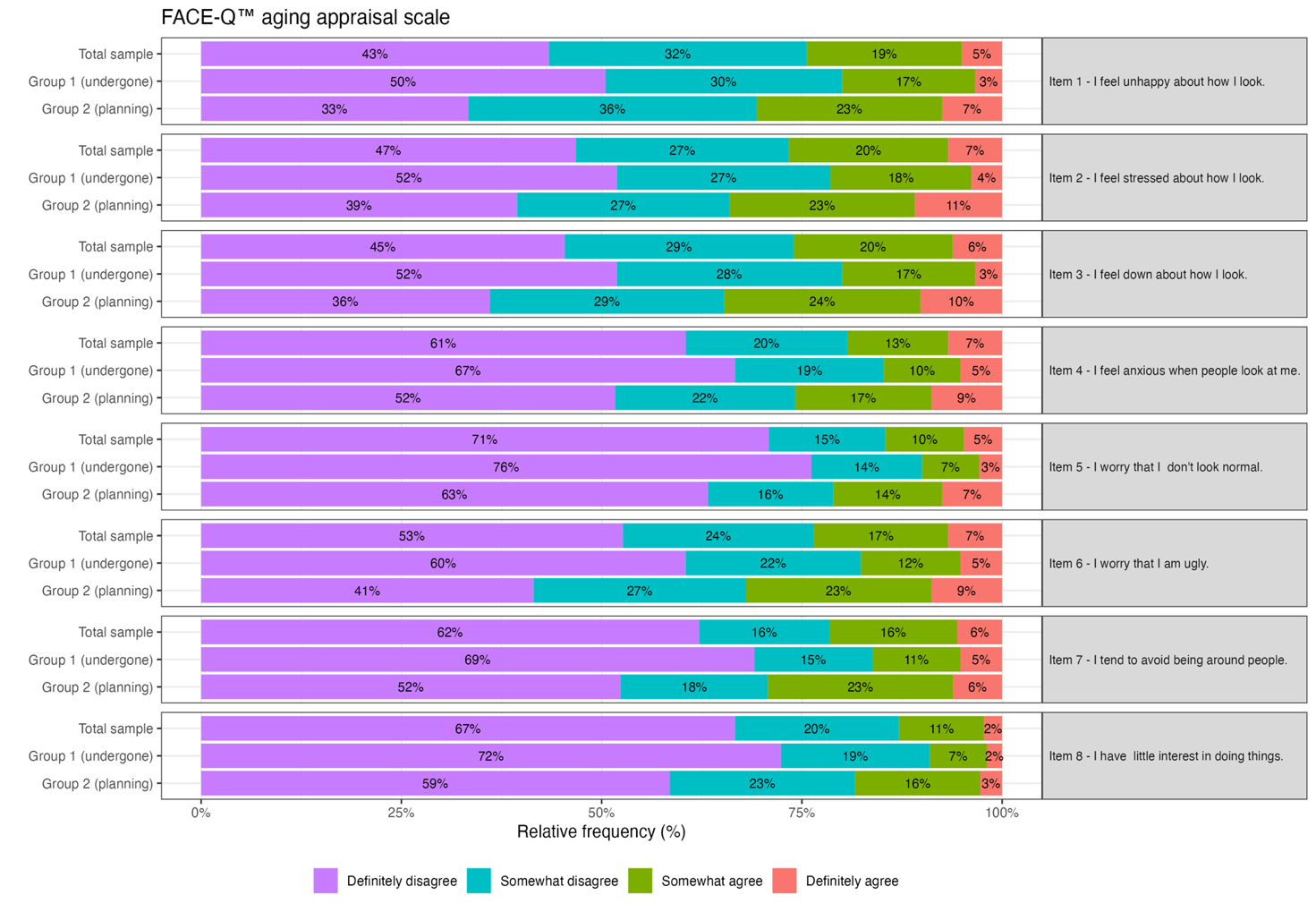


**Online resource 3. Distribution of the responses on the FACE-Q Psychosocial Distress scale**


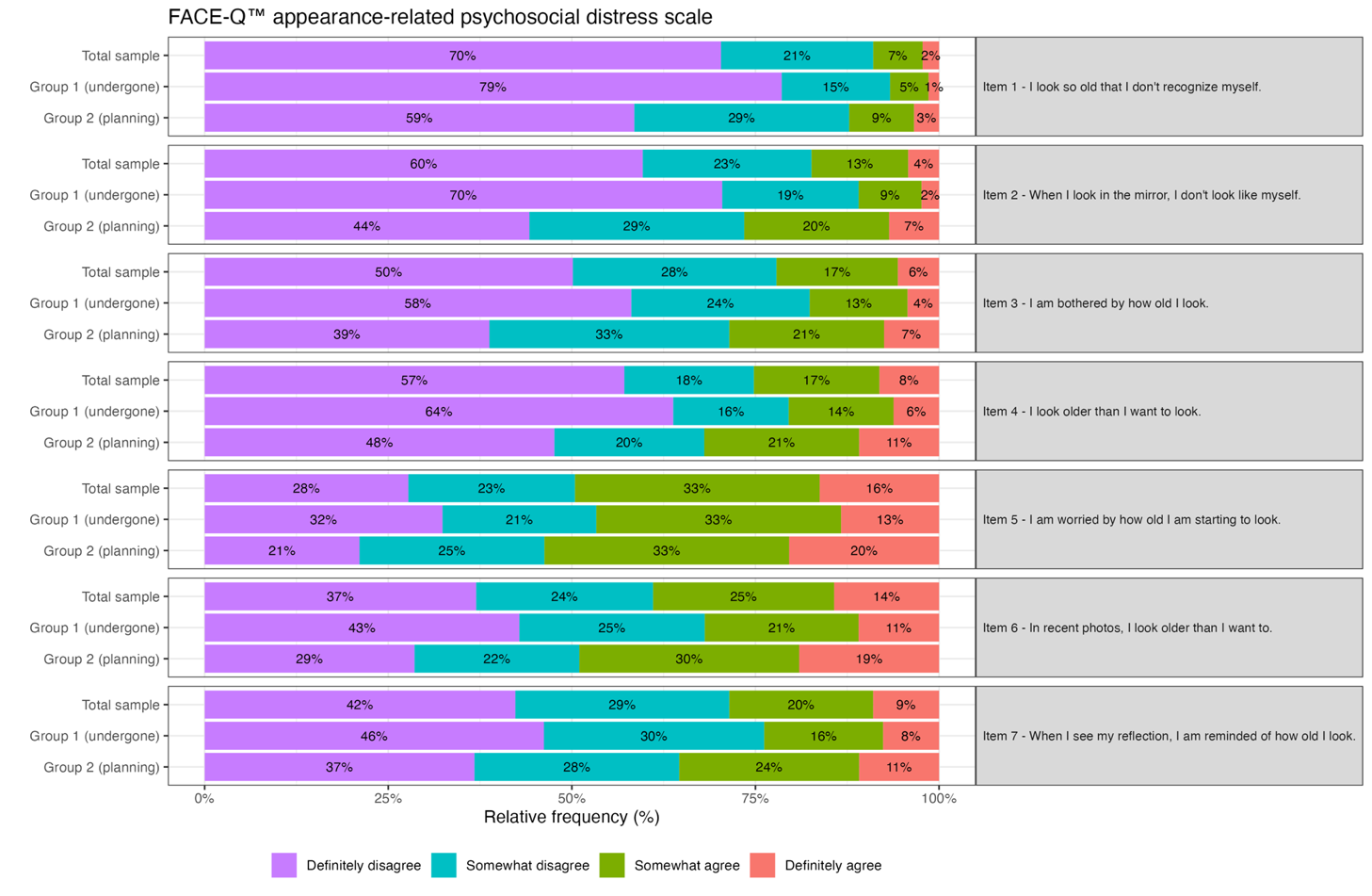

Supplement: Supplementary file 1 — Supplementary Material 1 [file 11136_2026_4313_MOESM1_ESM.docx]
